# Rectified directional sensing in long-range cell migration

**DOI:** 10.1038/ncomms6367

**Published:** 2014-11-06

**Authors:** Akihiko Nakajima, Shuji Ishihara, Daisuke Imoto, Satoshi Sawai

**Affiliations:** 1 Department of Basic Science, Graduate School of Arts and Sciences, University of Tokyo, Tokyo 153-8902, Japan; 2 Research Center for Complex Systems Biology, University of Tokyo, Tokyo 153-8902, Japan; 3 Department of Physics, School of Science and Technology, Meiji University, Kawasaki 214-8571, Japan; 4 PRESTO, Japan Science and Technology Agency, Kawaguchi-shi, Saitama 332-0012, Japan

## Abstract

How spatial and temporal information are integrated to determine the direction of cell migration remains poorly understood. Here, by precise microfluidics emulation of dynamic chemoattractant waves, we demonstrate that, in *Dictyostelium*, directional movement as well as activation of small guanosine triphosphatase Ras at the leading edge is suppressed when the chemoattractant concentration is decreasing over time. This ‘rectification’ of directional sensing occurs only at an intermediate range of wave speed and does not require phosphoinositide-3-kinase or F-actin. From modelling analysis, we show that rectification arises naturally in a single-layered incoherent feedforward circuit with zero-order ultrasensitivity. The required stimulus time-window predicts ~5 s transient for directional sensing response close to Ras activation and inhibitor diffusion typical for protein in the cytosol. We suggest that the ability of *Dictyostelium* cells to move only in the wavefront is closely associated with rectification of adaptive response combined with local activation and global inhibition.

## Abstract (web summary)

Cell migration is regulated by spatial and temporal information, but how the two are integrated is not well understood. Here, Nakajima *et al*. use dynamic microfluidics gradients to show that Ras activation at the leading edge of *Dictyostelium* is suppressed when chemoattractant concentration decreases over time.

## Graphical abstract

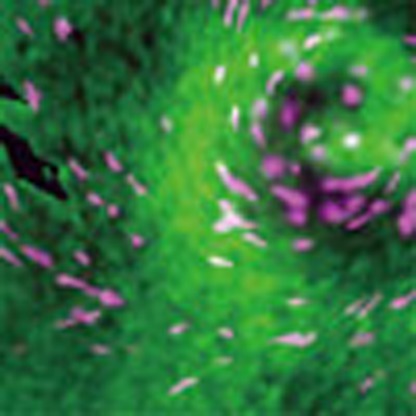

Long-range directional cell migration during embryonic development^1^,^2^ and wound-healing^3^ is directed by gradients of attractant cues that are often dynamic and self-enhancing^4^,^5^. In *Dictyostelium* and neutrophils, localized activation of small guanosine triphosphatases (GTPases) and their downstream effectors such as phosphoinositide-3-kinase (PI3K) recruit signalling molecules at the cell cortex to form the leading edge^6^,^7^. It is widely accepted that cells sense direction by comparing the attractant concentrations across the cell body^8^. Such spatial sensing, however, constitutes a challenge in long-range migration, because the attractant gradient must be sustained over long distances, and the cells must be able to sense a wide range of gradient steepness in the background of various mean concentrations. Aggregation of *Dictyostelium discoideum* appears to have partly solved this problem by self-enhancing cell-to-cell relay of chemoattractant cyclic AMP (cAMP) in the form of non-dissipating waves. However, because gradient reverses during the wave passage, it remains unclear how cells avoid futile back-and-forth movement^9^,^10^,^11^. This is the so-called ‘back-of-the-wave’ problem in *Dictyostelium* cell aggregation.

Chemotaxis of *D. discoideum* amoebae is mediated by G-protein coupled receptor signalling with multiple redundant pathways; target of rapamycin complex 2 (TORC2), PI3K, phospholipase A2 and guanylyl cyclases^7^,^12^. Localized activation of the small GTPase Ras at the leading edge of migrating cells constitute one of the earliest events of the symmetry breaking^13^. The null-mutants of Gbeta display no chemotaxis^14^, and Ras activation is completely abolished^15^. Multiple guanine-nucleotide-exchange factors (GEFs) and GTPase-activating proteins (GAPs) that regulate conversion between the GTP- and guanosine diphosphate-bound form of Ras have been identified^15^,^16^,^17^,^18^. While heterotrimeric G protein is activated non-adaptively as evidenced by the persistent dissociation between Gbeta and Galpha subunit^19^, activation of Ras^13^ as well as their downstream targets such as PI3K^13^ are adaptive, meaning their activities return to the pre-stimulus level under spatially uniform persistent stimulation. The two protein kinase B isoforms in *Dictyostelium* are regulated by TORC2 and PI3K^17^,^20^,^21^, and their null-mutants are heavily impaired in their chemotactic ability^22^. Protein kinases B suggest possible links to cell motility as their targets include Talin, RhoGAPs and PI5kinase^20^. While cells genetically and pharmacologically suppressed entirely of TORC2, PI3K, PLA and guanylyl cyclases are heavily impaired in chemotaxis, Ras activation was intact^12^. Taken together with the fact that *rasC*− cells expressing the dominant-negative form of RasG is strongly impaired in chemotaxis^15^, heterotrimeric G-protein and its downstream Ras activation are thought to form the basis of symmetry breaking in *Dictyostelium* chemotaxis^12^,^13^,^15^. However, since most studies are conducted in a stationary gradient or abrupt application of gradient using a micropipette, how their dynamics are dictated by temporally varying gradients remains poorly understood.

Since the stimulus experienced by the aggregating cells *in vivo* is in the simplest form of near sinusoidal wave, the *Dictyostelium* system serves as an ideal model to dissect how migrating cells in general process dynamically changing gradient information of more complexities. Two main hypotheses have been proposed to resolve the ‘back-of-the-wave’ problem. The most prevalent idea is that the cells become insensitive for a certain time period after exposure to the stimulus^9^,^10^,^11^,^23^. The molecular circuitry of chemotaxis and motility in *Dictyostelium* includes excitatory feedback modules^24^,^25^,^26^,^27^, thus in theory, refractoriness associated with the excitability can explain the directional movement in the travelling wave stimulus^27^. However, single-cell level assays of localized phosphatidylinositol (3,4,5)-trisphosphate (PIP3) synthesis^28^, chemotactic cell movement^29^ as well as tracking of isolated cells in the vicinity of aggregating streams^30^ showed no evidence for refractoriness in directional sensing.

Alternatively, cells may be employing a mechanism that discriminates temporally increasing and decreasing chemoattractant concentrations. Perfusion studies have shown that cell motility increases in spatially uniform and temporally increasing cAMP concentrations, however, not in decreasing cAMP concentrations^31^. While such a property suggests cell movement should slow down in the waveback, it was not clear why the cells did not reorient^31^. Earlier works that further addressed this problem by studying chemotaxis in temporally changing cAMP gradients yielded conflicting results^32^,^33^,^34^,^35^. While some works^32^,^36^ indicated that cells ascend the concentration gradient irrespective of the temporal change, others^33^,^34^,^35^,^37^ suggested that chemotaxis was suppressed when the cAMP concentration was decreasing in time. Controlling the gradients in time using a pressurized point source requires skilled manoeuvering of the micropipette^18^,^28^,^32^. Approaches using gradient chambers are also subjected to trial-to-trial variations, as they are based on passive diffusion in conjunction with either concentration changes at the source^34^,^35^, enzymatic degradation^33^ or manual positioning of attractant reservoirs^36^. The apparent discrepancies between these earlier works may have arisen from the fact that the basal levels and the gradient profiles were not fully defined, much less their time constants.

Recent advances in microfluidics have seen techniques that allow more accurate and rapid control of concentration gradients in time and space^38^,^39^. Continuously applied flow of attractant in combination with Percoll density gradients^40^ supports mechanically stable concentration gradients that can be monotonically increased or decreased in time, however, gradient reversal is difficult by design. The dual-layer pyramidal mixer^41^ does allow orientation reversal, however, not without introducing unwanted transients. These approaches are not easily compatible with the travelling wave stimulus, where precise displacement of a set of continuous up and down gradients is required. More recently, laminar flows from three independent inlets were combined and focused to generate gradients that could be varied continuously in time^39^. Unlike other techniques described above, gradient generation based on flow-focusing supports continuously changing gradients with finely controlled time constants and concentration range. In this study, we extend the flow-focusing approach to create bell-shaped gradients that can be displaced continuously in space to emulate travelling wave stimulus. By combining quantitative live-cell imaging analysis and dynamically controlled gradients, we elucidate how spatio-temporal information of the extracellular chemoattractant concentrations is encoded at the level of Ras activation. Furthermore, from mathematical analysis, we explore how an adaptive feedforward network can implement a rectifying circuit that filters out input signals based on temporal information.

## Results

### Cell movement in cAMP waves *in vivo* and *in vitro*

**Figure 1:**
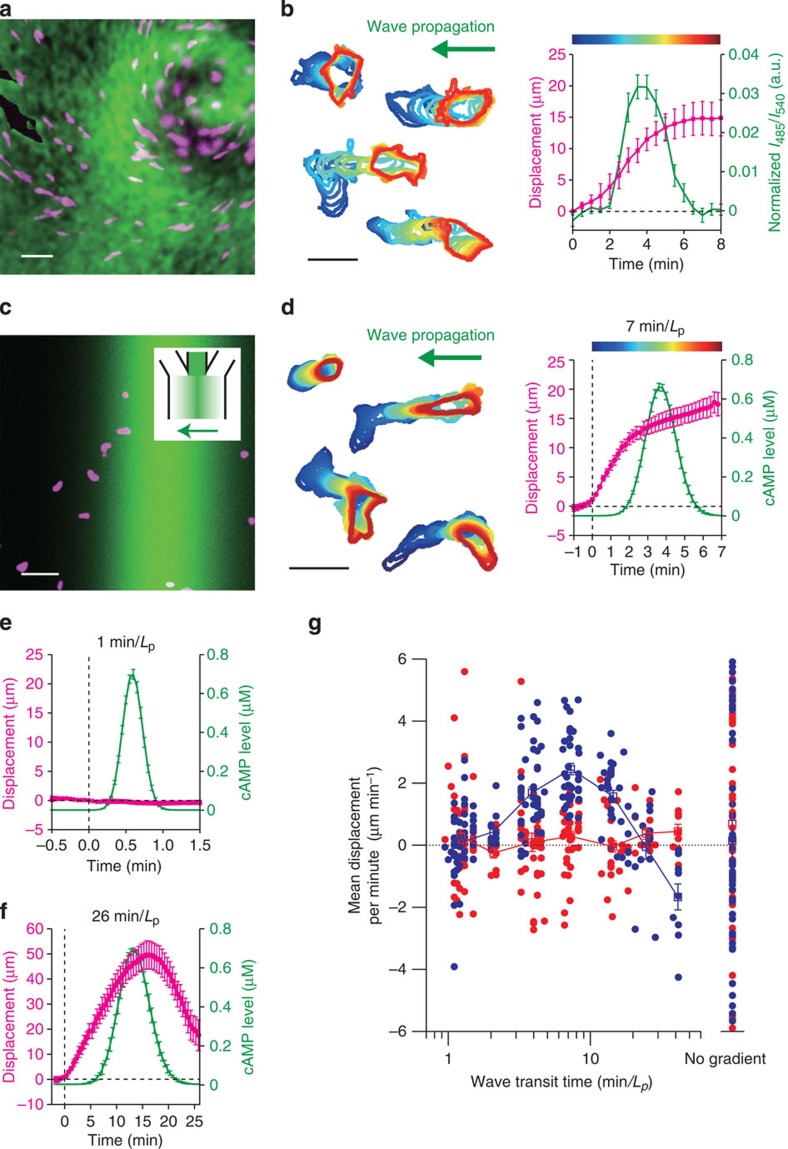
Chemotaxis in natural and artificial travelling waves of the chemoattractant cAMP. (**a**,**b**) cAMP waves and cell movement in the aggregation field. A frame-subtracted image of cytosolic cAMP (**a**, green; Epac1-camps^43^,^66^) and a fraction of cells co-expressing RFP for cell tracking (**a**; magenta) (see Methods). Cell contours (**b**; left panel) and cell displacements (right panel; *n*=7). Colours (left panel) represent the phase of cAMP waves (right panel). (**c**) A snapshot of an imposed wave (**c**, green; fluorescein) and cells (**c**; magenta). A schematic of the infusing flows (**c**; inset). (**d**–**f**) Cell displacements towards the cAMP pulse of 7 min per *L*_p_ (**d**, *n*=21), 1 min per *L*_p_ (**e**, *n*=16) and 26 min per *L*_p_ (**f**, *n*=6) duration. Time *t*=0 indicates the point at which the stimulus concentration exceeded a threshold (0.01% of the maximum) (see Methods). (**g**) Average velocity of cell migration parallel to the direction of wave propagation (**g**, *n*=199; blue). The velocity is positive in the direction against the incoming wave. The average cell velocity orthogonal to the propagating direction (**g**; red; control). The average from 4-min mock fluorescein waves (in the absence of cAMP (*n*=10) and 1 nM spatially uniform background cAMP (*n*=39)) (**g**, no gradient). Error bars indicate s.e.m. Scale bars; 50 μm (**a**,**c**) and 20 μm (**b**,**d**).

The relation between the cAMP waves and cell movement has been conventionally estimated from the periodic changes in the light scattering caused by the cell-shape change^9^,^30^. Because such analyses failed to separate changes in cell motility and the chemoattractant concentrations, we first revisited this aspect by employing a more direct measurement of cAMP. The oscillations of intra- and extracellular cAMP concentrations occur synchronously^42^, and the changes in the level of intracellular cAMP serve as a good indicator of the cAMP-induced cAMP relay in the chemoattractant field^43^. Figure 1a shows a snapshot of the cAMP waves and cell migration in the aggregation field (Supplementary Movie 1). Cells moved directionally in the wavefront, however, no reverse movement was observed in the waveback (Fig. 1b). Thus the ‘wave paradox’^9^,^10^,^11^ remains in defiance to the gradient-sensing paradigm, which nonetheless bases its claim on many other experimental observations in *Dictyostelium*^44^.

To test whether the rectified motion originates from hidden cues such as asymmetry in the profile of extracellular cAMP concentrations, cell–cell contacts or other cofactors known to affect chemotaxis^45^,^46^, we studied isolated cells under artificially generated cAMP waves. Waves of a size similar to natural waves (*L*_p_=850 μm in width) that are spatially symmetric were generated by flow-focusing in a microfluidic channel^39^ (Fig. 1c; Supplementary Fig. 1). When cAMP waves were applied at a period of 7 min per *L*_p_, cells migrated directionally towards the incoming waves (Fig. 1d, see also Supplementary Movie 2) just as they would in the aggregation field. The speed of cell migration increased in the wavefront and reached 7 μm min^−1^ comparable to that during the early stage of cell aggregation (Supplementary Fig. 2). For fast waves (<2 min per *L*_p_), no migration was observed (Fig. 1e). For slow waves (>10 min per *L*_p_), cells first moved towards the incoming wave but then reversed their migratory direction in the waveback (Fig. 1f). As summarized in Fig. 1g, cells migrated directionally towards the incoming waves only when the transit time was between 3 and 10 min per *L*_p_, which overlaps well with the time period of the native cAMP waves^47^,^48^. These observations indicate that the rectified movement is not absolute and that the temporal-scale rather than the spatial asymmetry of the wave is critical. Interestingly, for slow waves, the mean displacement became negative most likely due to the ‘Doppler’ effect (see Supplementary Note; see also Supplementary Fig. 6).

### Ras activation in dynamically changing gradients

**Figure 2:**
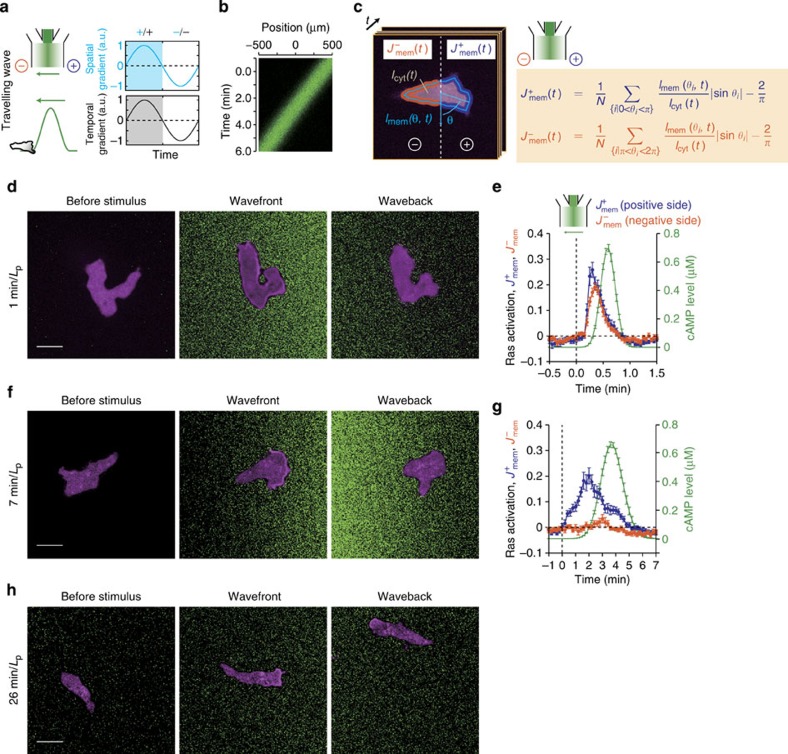
Ras response in the travelling wave stimulus is time dependent. (**a**,**b**) Propagating wave stimulus. (**c**) A schematic of quantification of RBD membrane localization (see Methods for details). (**d**–**h**) RBD translocation during the travelling wave stimulus; transit time 1 min per *L*_P_ (**d**,**e**), 7 min per *L*_P_ (**f**,**g**) and 26 min per *L*_P_ (**h**). Representative confocal images of spatially localized Ras activity (RFP-RBD; magenta) induced by the artificial cAMP wave stimuli (fluorescein; green) (**d**,**f**,**h**). Time series of RBD translocation to the positive side (blue) and the negative side (orange) (**e**,**g**). Time *t*=0 indicates the point at which the stimulus concentration exceeded a threshold (0.01% of the maximum). A half side of the cell boundary facing the right-hand side of the chamber was defined as the positive side (see Methods). For the wave stimulus of 26 min per *L*_P_ transit time, active cell movement in the *z* axis direction prevented us from obtaining reliable time series. Data are averages over *n*=16 (**e**) and 21 (**g**)±s.e.m. (error bars). Scale bar, 10 μm.

To gain insights on the selective movement towards the cAMP wave, directional sensing under a single pulsatile wave was quantified by monitoring translocation of fluorescent protein fused to a Ras-binding domain (RBD), which binds to the activated form of Ras^12^,^13^,^49^,^50^ (Fig. 2a–c). For transit time of <2 min per *L*_p_, translocation of RBD to the plasma membrane occurred uniformly (Fig. 2d,e). For passage time of 7 min per *L*_p_, RBD translocated only towards the side facing higher cAMP concentrations during the first 2 min (Fig. 2f,g; Supplementary Movie 3). During the following 5 min while the level of membrane-bound RBD returned to the pre-stimulus level, there was no reversal in the RBD distribution. For slow waves, RBD localized in the direction facing higher cAMP concentrations both in the wavefront and the waveback (Fig. 2h), consistent with the reversed cell movement in the waveback (Fig. 1f).

**Figure 3:**
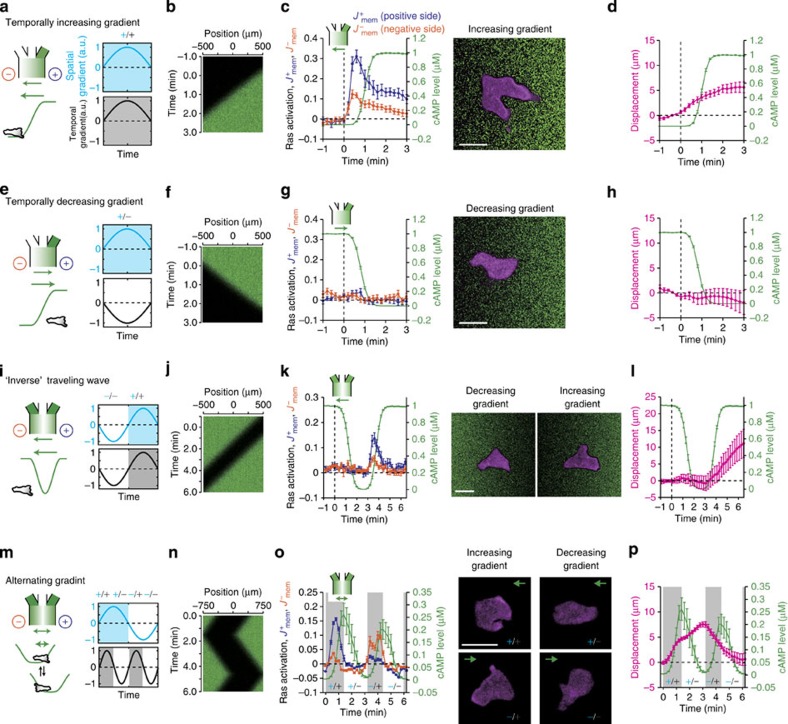
Chemotaxis and directional sensing in dynamically changing gradients are rectified by temporal information. (**a**–**d**) Temporally increasing gradient. (**e**–**h**) Temporally decreasing gradient. (**i**–**l**) Inverse wave. (**m**–**p**) Alternating gradient. The sign of the space (blue)/time (grey) derivatives of the stimulus are indicated by ‘+’ and ‘−’ (**o**,**p**). Schematics (**a**,**e**,**i**,**m**), and the space–time plot (**b**,**f**,**j**,**n**) of the stimulus. RBD localization in the positive and negative direction (**c**,**g**,**k**,**o**; left panel; blue and orange) and representative confocal images (**c**,**g**,**k**,**o**; right panels). Cell displacement (**d**,**h**,**l**,**p**; magenta). Time series of the extracellular cAMP level (**c**,**d**,**g**,**h**,**k**,**l**,**o**,**p**; green). Time *t*=0 indicates the point at which the stimulus concentration was above (**c**,**d**) or below (**g**,**h**,**k**,**l**) a threshold (0.01% of the maximum for **c** and **d**; 99.5% for **g**, **h**, **k** and **l**). Data were averages over *n*=13 (**c**,**d**), 22 (**g**,**h**), 21 (**k**,**l**) and 6 (**o**,**p**) cells. Error bars indicate s.e.m. Scale bar, 10 μm.

To delineate possible mechanisms of rectified movement, we carried out a systematic survey on Ras activation (RBD translocation) and cell movement under all possible combinations of sign in the space and the time derivatives (Fig. 3). First, temporally increasing (Fig. 3a,b) or decreasing (Fig. 3e,f) spatial gradients were imposed by moving a gradient at a constant speed. As expected, in temporally increasing spatial gradients, Ras was activated at the cell edge facing the higher concentrations of cAMP (Fig. 3c), and cells migrated accordingly (Fig. 3d). In contrast, no Ras activation (Fig. 3g) nor directional cell migration (Fig. 3h) was detected in temporally decreasing gradients. Unlike the back of the travelling wave that cannot be experienced without first being exposed to the wavefront, here the cells were first allowed to fully adapt to spatially uniform cAMP concentrations (>5 min) before experiencing the gradient. Thus suppression of directional sensing appears not to require history of the past gradient and cell polarity.

**Figure 4:**
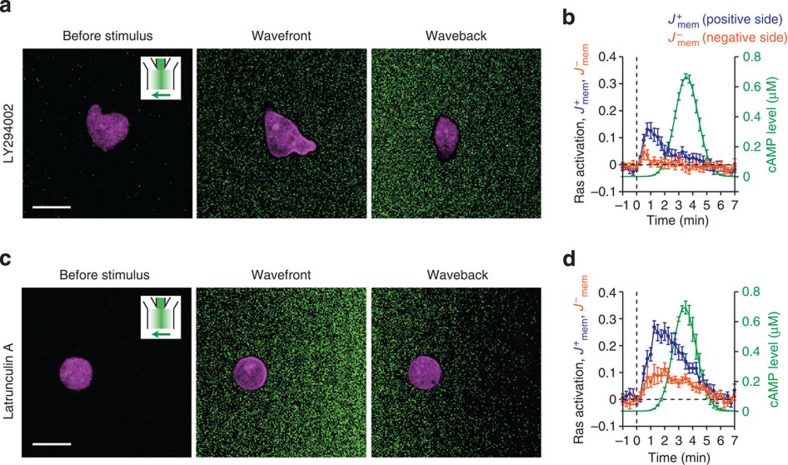
The rectified Ras response does not require feedback from downstream PI3K/F-actin signaling. (**a**–**d**) RBD translocation to 7 min per *L*_P_ wave stimulus in pharmacologically treated cells; 50 μM LY294002 (**a**,**b**) and 5 μM Latrunculin A (**c**,**d**). Representative confocal images of Ras activity (RFP-RBD; magenta) and the wave stimuli (fluorescein; green) (**a**,**c**). Time series of RBD localization at the positive side half (blue) and the negative side half (orange) of the cell membrane (**b**,**d**). Extracellular cAMP levels (**b**,**d**; green). Time *t*=0 was defined as the time point at which the stimulus concentration exceeded a threshold (0.01% of maximum concentration). Data are averages±s.e.m. (error bars) over *n*=10 (**b**) and 10 (**d**). Scale bar, 10 μm.

The importance of temporal information was further vindicated by the ‘inverse wave’ (Fig. 3i,j), where the signs in space and time derivatives were inverted with respect to the normal wave. Again, there was no Ras activation (Fig. 3k) and no net cell movement (Fig. 3l) in the temporally decreasing gradient. Not until the cAMP concentrations started to increase in the second slope (Fig. 3k,l; *t*>2.6 min), did Ras activation (Fig. 3k) and cell migration (Fig. 3l) become detectable. Moreover, when the temporal gradient was reversed by retreating the inverse wave while retaining the orientation of the spatial gradient (Fig. 3m,n), directional sensing at the level of Ras and cell movement were again suppressed (Fig. 3o,p; non-shaded time-windows). Although there was slight retention of directional movement (Fig. 3p; see also Fig. 1d) and Ras activity (Fig. 3c) after the rising phase, Ras activity was never sustained under temporally decreasing gradients (Fig. 3o; see also Fig. 2g). Furthermore, although treatment with the PI3K inhibitor LY294002 (LY) diminished the peak amplitude of the response, it had minimal effect on the selectivity of the response to temporally increasing gradients (Fig. 4a,b; Supplementary Fig. 3a–f). Similarly, in cells immobilized by Latrunculin A treatment, although suppression of Ras activation at the rear of the cells became less prominent, the response itself was still selectively observed for temporally increasing stimuli (Fig. 4c,d; Supplementary Fig. 3g–j), suggesting that the downstream excitable feedback circuit^25^,^26^,^51^ is not necessary for the rectification. These observations further indicate that the transient response is suppressed for temporally decreasing mean concentrations of the chemoattractant^31^,^33^ and that this occurs at the level of or upstream of Ras.

**Figure 5:**
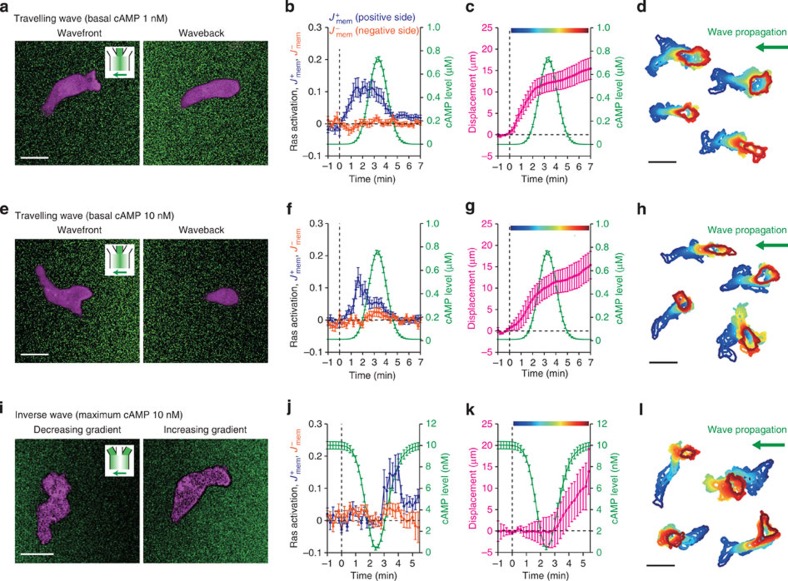
Ras activation and cell migration are rectified in the normal and inverted travelling wave stimuli with elevated background levels of cAMP. (**a**–**h**) Time series of the wave stimulus with elevated background levels of cAMP. The basal cAMP levels were 1 nM (**a**–**d**) and 10 nM (**e**–**h**). (**i**–**l**) The concentration of inverted wave stimulus was 10 nM at the maximum. Representative confocal images of localized Ras activation (RFP-RBD; magenta) and the spatial profile of the cAMP gradients (fluorescein; green) (**a**,**e**,**i**). Time series of RBD localization to the positive side (*J*^*+*^_mem_, blue) and the negative side (*J*^−^_mem_, orange) (**b**,**f**,**j**), cell displacement (**c**,**g**,**k**; magenta) and contours of representative cells (**d**,**h**,**l**). Extracellular cAMP levels (**b**,**c**,**f**,**g**,**j**,**k**; green). Time *t*=0 was the time point at which the stimulus concentration was above (**b**,**c**,**f**,**g**) or below (**j**,**k**) a threshold (0.01% of maximum concentration for **b**, **c**, **f** and **g**, and 99.5% for **j** and **k**). Colours of cell contours (**d**,**h**,**l**) correspond to the phase of the applied cAMP waves (**c**,**g**,**k**). Data plots are averages±s.e.m. (error bars) over *n*=19 (**b**,**c**), 20 (**f**,**g**), 8 (**j**) and 15 (**k**) cells. Scale bar, 10 μm (**a**,**e**,**i**) and 20 μm (**d**,**h**,**l**).

We should note that there appears to be an additional effect from cell memory as evidenced by extended cell migration in the wake of wave stimulation (Fig. 1d right panel *t*>3 min). Interestingly, there was almost no detectable RBD translocation during the later phase of this movement (Fig. 1d *t*>5 min), indicating that, once established, polarity can be maintained in the absence of marked Ras activation. To test whether such memory effect plays a role in the suppression of directional sensing, we studied travelling wave stimuli with elevated background levels of cAMP (Fig. 5a–h). Under such conditions, there was marked cell polarization and movement in random direction prior to gradient exposure. As soon as the cells were exposed to the rising wavefront, RBD localized to the side facing the higher concentrations of cAMP, and cells reoriented and moved in the correct ascending direction. Because travelling wave stimulus on top of 10-nM background cAMP still elicited the rectified response (Fig. 5e–h), lack of gradient sensing in the decreasing gradient of the inverse wave down from 10 nM cAMP (Fig. 5i–l; *t*<2.4 min) is difficult to explain by the memory effect of absolute cAMP concentrations. These results further demonstrate that temporal increase in the chemoattractant concentrations is essential for Ras activation and reorientation.

### Rectified response in a directional sensing model

**Figure 6:**
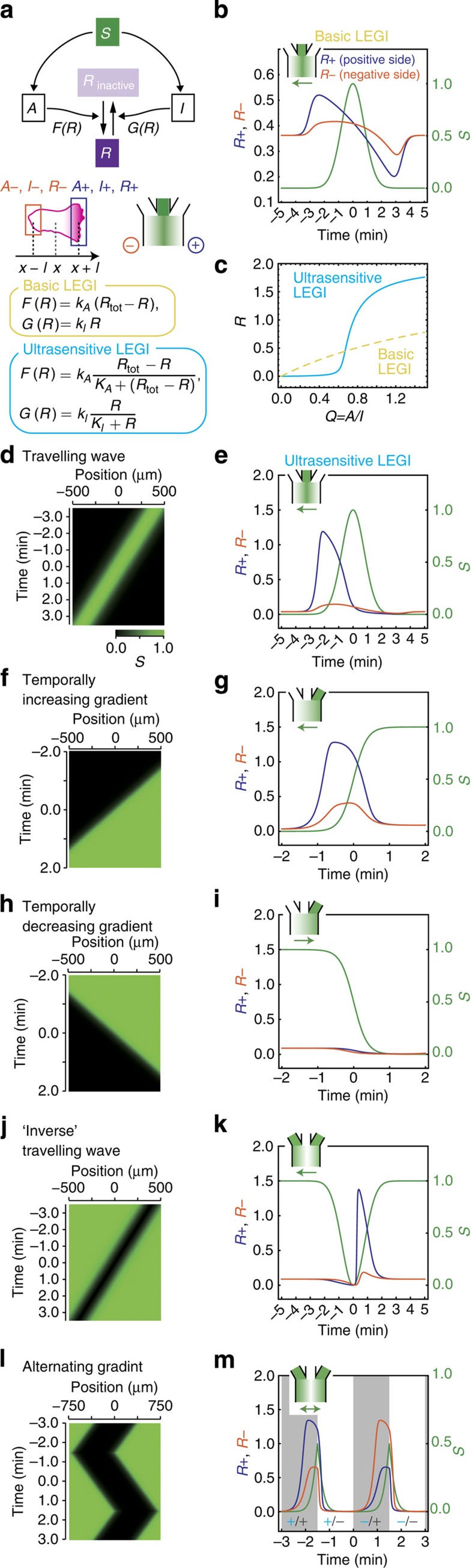
An ultrasensitive LEGI circuit implements rectification. (**a**) A schematic of the incoherent feedforward circuit in the LEGI model. One-dimensional space was considered where a cell of length 2*l* was positioned at *x*=0 (see Methods for details). Space was discretized and coarse-grained so that variables *A*, *I* and *R* were considered only at the ends of a cell; denoted by *A*_+_, *A*_−_, *I*_+_, *I*_−_, *R*_+_ and *R*_−_. The ‘+’ and ‘−’ signs indicate the direction in the chamber. (**b**) Numerical simulations of the basic LEGI equation for the travelling wave stimulus. The output in the positive side (*R*_+_, blue) and the negative side (*R*_−_, orange) of a cell. In travelling wave stimulus, the positive side faces the rising side of the incoming wave. (**c**) The resting state of *R* plotted against the change in the activator/inhibitor ratio *Q*. (**d**–**m**) Numerical simulations of the ultrasensitive model; travelling wave stimuli (**d**,**e**), temporally increasing (**f**,**g**) and decreasing (**h**,**i**) gradients, inverse travelling wave (**j**,**k**) and alternating gradient (**l**,**m**). The sign of the space (blue)/time (grey) derivatives of *S* are indicated by ‘+’ and ‘−’ (**m**). See Supplementary Table 1 for model parameters.

Many of the essential properties of directional sensing and the adaptive Ras activation have been understood from the framework of the so-called local excitation global inhibition (LEGI) model^52^ and its variants^44^,^50^,^53^ (Fig. 6a). A detailed modelling has been proposed that maps this scheme primary to the regulation of Ras between its GTP- and guanosine diphosphate-bound forms^50^. The basic LEGI framework assumes two mediators; activator ‘*A*’ and inhibitor ‘*I*’ of the output *R*. ‘*A’* and ‘*I’* are both positively regulated by the input signal ‘*S*’. For spatially uniform input, the model is described by the following equations:




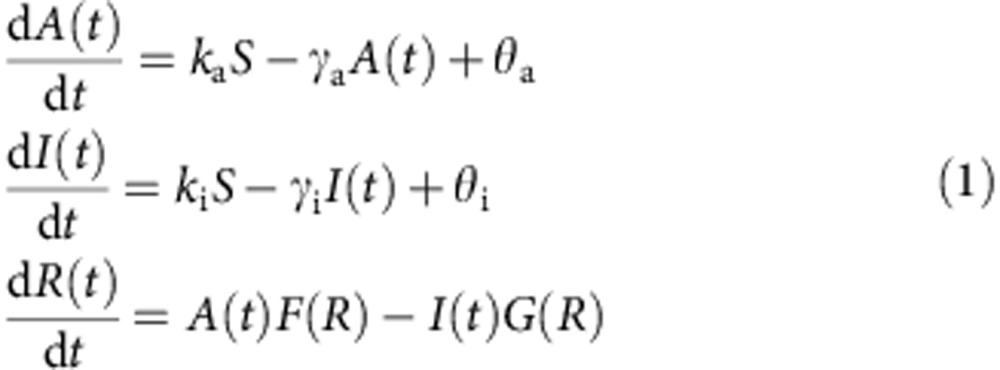




where *F*(*R*) and *G*(*R*) are functions shown in Fig. 6a (yellow box; basic LEGI). In the first and second equations, *k*_a_, *k*_i_ and *γ*_a_, *γ*_i_ determine the rate of increase and decrease of the activator ‘*A’* and the inhibitor ‘*I’* molecules, respectively. *θ*_a_ and *θ*_i_ are basal activation rates that determine imperfectness of adaptation. Because we observed that adaptation of RBD translocation was slightly imperfect (Supplementary Fig. 4), here *θ*_a_ and *θ*_i_ are assumed to be small but non-zero. The first and second terms in the third equation describe activation and deactivation of *R* by *A* and *I*, respectively. Upon spatially uniform increase in ‘*S*’, due to a higher rate of activation, there would be a transient rise in the output ‘*R’* followed by its return to the pre-stimulus level by the action of the inhibitor ‘*I*’ (Supplementary Fig. 5a). Thus the model describes well the adaptive ‘temporal sensing’ property of the chemotactic response to spatially uniform stimuli. The other essential feature of the LEGI scheme is that the inhibitor ‘*I*’ diffuses fast and acts globally. Thus, for a stationary gradient stimulus, while the activator level mirrors the local receptor occupancy, the inhibitor level traces its average. Consequently, the ratio *Q*=*A*/*I*, which dictates the output *R*, would always transmit the relative difference in the input signal *S* from the background (Supplementary Note; see also Supplementary Fig. 5b). From this ‘spatial sensing’ property, intracellular gradient of the ratio *Q* within the cell faithfully mirrors the applied attractant gradient irrespective of the temporal change. Although a possible resolution to the ‘wave paradox’ may be provided for rapidly changing signals where the response transients could significantly deviate from the stationary state, the basic LEGI scheme nevertheless predicts symmetric responses in both wavefront and waveback (Fig. 6b; see Methods for equations). How would the discrepancy between the model prediction and the observed rectification be resolved?

**Figure 7:**
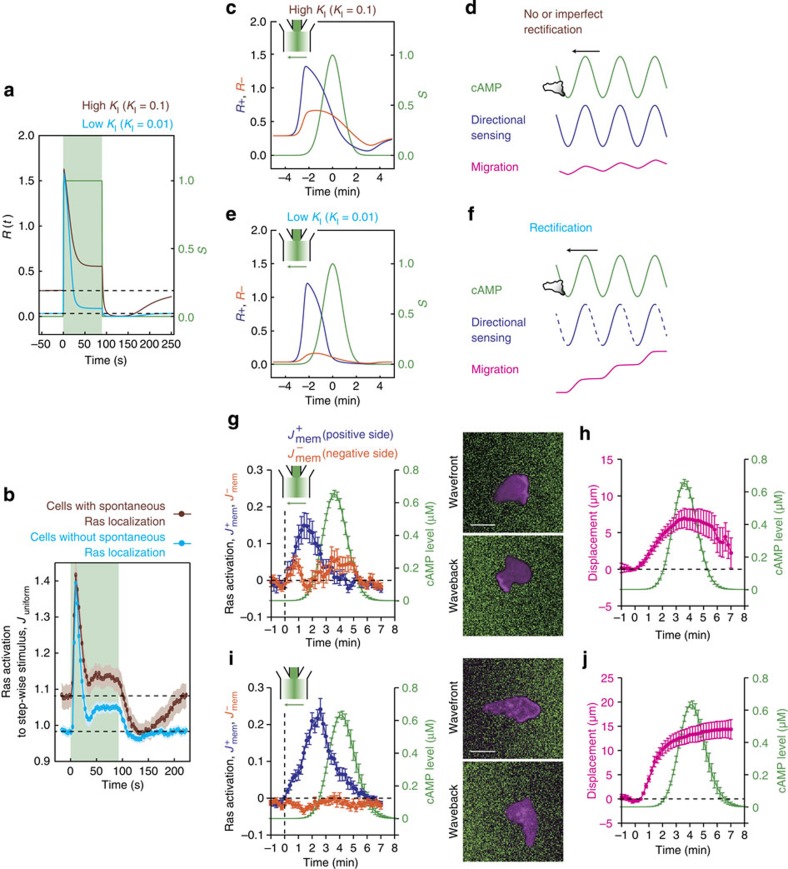
Asymmetry in the response to temporally increasing and decreasing stimulus. (**a**) Adaptive response to spatially uniform increase and decrease of the signal input (**a**, green line and shaded area) in the ultrasensitive LEGI model for high *K*_I_ (*K*_I_=0.1) (**a**, brown) and low *K*_I_ (*K*_I_=0.01) (**a**, cyan). (**b**) Ras response to spatially uniform increase and decrease of cAMP (between 0 and 1 μM cAMP; green shaded area) in weakly starved cells with (**b**, brown; *n*=24) or without (**b**, cyan; *n*=20) spontaneous RBD localization. (**c**–**f**) Directional sensing response in the ultrasensitive LEGI model for high *K*_I_ (*K*_I_=0.1) (**c**) and low *K*_I_ (*K*_I_=0.01) (**e**). Schematics of chemotactic response to traveling wave stimulus without (**d**) or with (**f**) rectification. (**g**–**j**) Ras response (**g**,**i**) and cell movement (**h**,**j**) in travelling wave stimulus in cells with (**g**,**h**; *n*=22) or without (**i**,**j**; *n*=19) spontaneous Ras localization. Time *t*=0 indicates the point at which the stimulus concentration exceeded a threshold (0.01% of the maximum). Error bars indicate s.e.m. Scale bar, 10 μm.

As a natural extension of the basic LEGI model, let us examine a case where the kinetics *F*(*R*) and *G*(*R*) follow the Michaelis–Menten form^54^ (Fig. 6a; cyan box) thereby equipping the LEGI circuit with an ultrasensitive transfer function^55^ (Fig. 6c; see also Supplementary Fig. 5k; Supplementary Note for analysis). The output response predicted from the model are now largely consistent with the observed Ras response for the travelling wave stimulus (Fig. 6d,e) as well as monotonically changing gradients (Fig. 6f–i), inverse waves (Fig. 6j,k) and alternating gradients (Fig. 6l,m). Owing to simplicity of the ultrasensitive model, the basis of rectification can be well described by the response to uniform stimulation. The basic LEGI model predicts a strong undershooting response for a spatially uniform and temporally decreasing stimulus (Supplementary Fig. 5a–c; refs 50, 52). In contrast, the ultrasensitive LEGI circuit does not respond to the temporally decreasing stimulus (Fig. 7a cyan; Supplementary Fig. 5d–f) due to strong suppression at work in the zero-order regime. This is in accordance with the observed changes in the level of membrane-bound RBD to increase and decrease in spatially uniform cAMP concentrations (Supplementary Fig. 4a; see also Fig. 7b cyan). Immediately after the release from the prolonged exposure to spatially uniform cAMP concentrations, Ras activity quickly recovered the pre-stimulus level without an undershoot (Supplementary Fig. 4a). Together with the apparent absence of membrane-bound RBD prior to stimulation (Fig. 2d,f,h), the results are in line with the perfect shutdown of the resting-state response in the ultrasensitive regime.

The ultrasensitive model reduces to the basic LEGI model in the limit of high Michaelis–Menten constants (*K*_I_ in Fig. 6a), therefore predicts an undershooting response to a temporally decreasing uniform stimulus (Fig. 7a brown; high *K*_I_ (*K*_I_=0.1); and Supplementary Fig. 5g–j). Consequently, directional sensing occurs both in the wavefront and the waveback (Fig. 7c,d), in marked contrast to the rectified response at low *K*_I_ (Fig. 7e,f). To test the model predictions, we took advantage of a high occurrence of spontaneous Ras activation in weakly starved cells (≈40% total cells; Fig. 7b brown; *t*<0 min). In these cells, there was a transient undershoot of Ras activity upon release from a uniform cAMP stimulus (Fig. 7b, 100–200 s). Moreover, these cells sensed the gradient not only in the wavefront but also in the waveback (Fig. 7g), hence exhibited back-and-forth movement (Fig. 7h). These behaviours are in striking contrast to the more asymmetric response observed for weakly starved cells without spontaneous RBD localization (Fig. 7b cyan; Fig. 7i,j).

### Model prediction of the essential parameters

**Figure 8:**
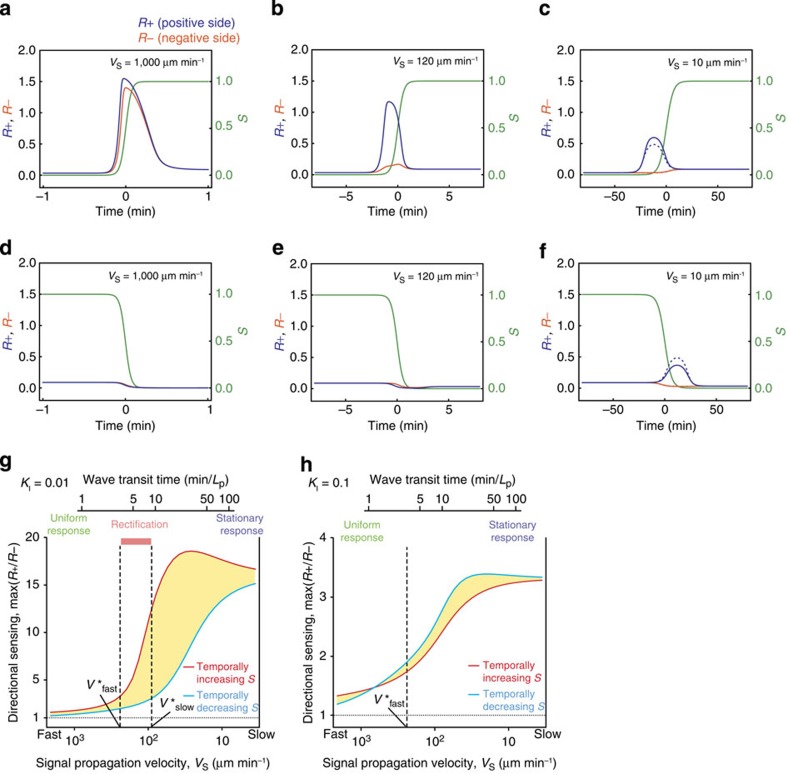
Rectified directional sensing can be attributed to the low-pass filter characteristics in the ultrasensitive circuit. (**a**–**f**) Time series from the numerical simulations in the ultrasensitive LEGI model (*K*_I_=0.01). Response in the positive (+) side (*R*_+_, blue) and the negative (−) side (*R*_−_, orange) of a cell to monotonic gradients that are increasing (**a**–**c**) or decreasing (**d**–**f**) in time. The spatial-temporal profiles of *S* were the same as in Fig. 6f–i. Monotonic gradients move from right to left (in the (−) direction) for temporally increasing gradients, and left to right (in the (+) direction) for temporally decreasing gradients. Hence, the positive (+) direction always faces higher concentrations of the monotonic gradients. The propagation velocity *V*_S_ for fast- (*V*_S_=1,000 μm min^−1^; **a**,**d**), intermediate- (120 μm min^−1^; **b**,**e**) and slow- (10 μm min^−1^; **c**,**f**) moving gradients. Dashed lines (**c**,**f**) indicate the analytically obtained stationary states of *R*_+_ and *R*_−_ for a given signal gradient evaluated at each time point. (**g**,**h**) Maximum values of the ratio *R*_+_/*R*_−_ in response to temporally increasing (red) and decreasing (cyan) spatial gradients in the ultrasensitive LEGI model at *K*_I_=0.01 (**g**) and *K*_I_=0.1 (**h**). Corresponding wave transit time for *L*_P_=800 μm is indicated (**g**,**h**; top *x* axis label). Asymmetry in directional sensing to temporally increasing and decreasing stimulus is highlighted by the shaded regions. The characteristic velocity, V*_fast_ and V*_slow_ are indicated in black dashed lines. See Supplementary Table 1 for model parameters.

The origin of the timescale dependence (Figs 1g and 2) can be identified by analysing the behaviour of the ultrasensitive LEGI circuit in moving gradients (Fig. 8; see also Supplementary Fig. 6 for travelling wave stimulus). For low *K*_I_ (*K*_I_=0.01), we see that the response can be classified qualitatively into three regimes depending on the propagation velocity *V*_S_ (Fig. 8). When *V*_S_ is large (*V*_S_=1,000 μm min^−1^), there is a spatially uniform increase in the output *R* in temporally increasing gradients (Fig. 8a). In contrast, *R* remains at the basal level in temporally decreasing gradients (Fig. 8d). For small *V*_S_ (*V*_S_=10 μm min^−1^), the output *R* is always greater on the side facing higher concentrations of *S* irrespective to its time derivative (Fig. 8c,f). At an intermediate signal velocity (*V*_S_=120 μm min^−1^), the output *R* rises only in the temporally increasing gradients, and this is spatially restricted towards the side facing higher concentration of *S* (Fig. 8b,e).

The upper bound of signal velocity provides us with an estimate of diffusion constant of the inhibitor ‘*I*’ independent of the model details. In order for a cell to sense the moving gradient of the chemoattractant, time required for the inhibitor to spread out within a cell (2*l*)^2^/2*D* must be shorter than the time lag of signal detection between the two ends of a cell, 2*l/V*_S_; *l* is the cell radius (Fig. 6a) and *D* is the diffusion coefficient of the inhibitor. In other words, the inhibitor that initially increased at the cell front must immediately diffuse intracellularly and reach the other end of a cell before the stimulus does, otherwise the response transients would be equal in magnitude between the two ends of the cell (see Fig. 8a). Hence, we obtain the upper bound for the propagation speed




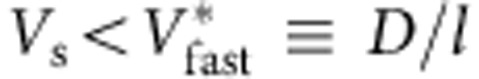




In principle, chemoattractant gradients travelling faster than this limit could only be perceived as spatially uniform stimulation. From the limit of directional sensing, we obtained V*_fast_≈240 μm min^−1^ (Figs 1g and 2) thus for cell radius *l*=7.5 μm, we estimate the diffusion constant of the inhibitor to be approximately *D*≈30 μm^2^ s^−1^, which matches well with those reported for green fluorescent protein (GFP) and GFP-tagged protein in the cytosol^56^,^57^. The result is suggestive of an inhibitor protein that shuttles between the plasma membrane and the cytosol as the higher mobility in the cytosol would dominate its diffusion. Although other mechanisms such as tension-based global inhibition^58^ cannot be ruled out, our analysis indicates that the diffusion process is sufficiently fast to meet the required global effect. While the analysis assumed activator diffusion to be negligibly small for the sake of mathematical analysis, the estimate and the overall model behaviours hold as long as the diffusion constant of the activator is more than one order of magnitude smaller (≤3 μm^2^ s^−1^; Supplementary Fig. 7a–c). Because membrane-bound protein diffusion falls well within this range, it may be that the activator molecule is more strongly sequestered to the plasma membrane.

On the other hand, the lower bound of signal velocity for the rectified migration readily reveals that the pulsatile response is essential for directional sensing in dynamically changing gradients. In a gradient *S*(*x*, *t*), a cell at the position *x*=0 at time *t* experiences *S*(+*l*, *t*) at the positive end (*x*=+*l*) and *S*(−*l*, *t*) at the negative end (*x*=−*l*). For slowly moving gradients (*V*_S_<V*_fast_), the concentrations of the activator and the inhibitor at both ends of a cell are approximately *A*_±_(*t*)≈*γ*_a_^−1^*k*_a_*S*(±*l*, *t−γ*_a_^−1^) and *I*_±_(*t*)≈*γ*_i_^−1^*k*_i_*S*_av_(*t*−*γ*_i_^−1^). Here, *S*_av_(*t*−*γ*_i_^−1^)=[*S*(+*l*, *t*−*γ*_i_^−1^)+*S*(−*l*, *t*−*γ*_i_^−1^)]/2 is the spatial average of the input signal. Hence,




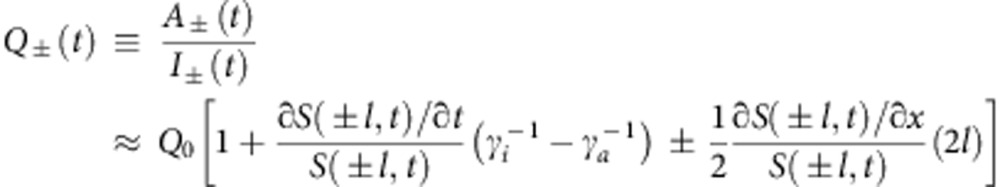




where *Q*_0_=*γ*_a_^−1^*k*_a_/*γ*_i_^−1^*k*_i_. The second and the third term represents the response in *Q*_±_ to temporal and spatial changes in *S*, respectively. In the slow limit of propagation speed, that is, stationary gradient, the second term vanishes, therefore *Q*_−_<*Q*_0_<*Q*_+_ always holds for a positive stationary gradient ∂*S*(±*l*, *t*)/∂*x*>0 (Supplementary Fig. 5l). In temporally decreasing gradients with non-zero propagation velocity, ∂*S*(±*l*, *t*)/∂*t*<0 thus again *Q*_−_ always satisfies *Q*_−_<*Q*_0_. Since rectification requires that changes in *Q*_−_ not be conveyed to downstream *R*, *Q*_+_>*Q*_0_ must be satisfied in order for a cell to sense the gradient direction. By combining these conditions and the relationship ∂*S*/∂*t*=−*V*_S_(∂*S*/∂*x*), we arrive at the lower bound for the rectified directional sensing




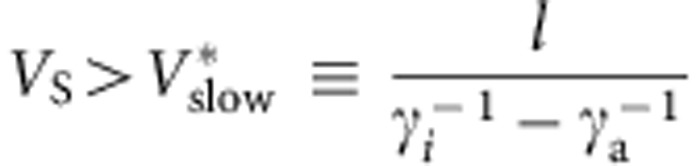




From our migration assays, V*_slow_≈90 μm min^−1^ (Fig. 1g) thus for a cell radius *l*=7.5 μm, we obtain 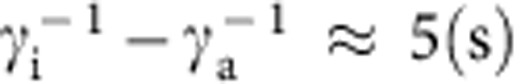, which is close to the observed transient of the RBD translocations.

Although chemotactic response in migratory cells is often characterized by the pulsatile response that peaks in the timescale of seconds, such as that observed here for Ras activation, its role in chemotaxis has not been well defined. The above inequality states that the gradient must travel a distance longer than the cell size within the time-window of the transient response in *R* (but no faster than the upper bound V^*^_fast_). Otherwise, the time-window would be long enough to support slow relaxation dynamics of *R* to its stationary state (that is, stationary spatial sensing scheme), which does not discriminate temporally negative and positive changes in the chemoattractant concentrations. The current analysis corrects the misconception in the field that cells must be in a refractory period of chemotactic response for a few minutes while they experience the waveback gradient. Although refractoriness associated with excitable dynamics could explain rectified movement towards the propagating waves, the reported refractory periods are 16.5 s for Ras^26^ and 30 s for PI3K^59^, which are both too short to explain the lack of response in the waveback. The rectified adaptive sensing predicts a spatially localized response transient of a seconds timescale, not minutes.

To summarize, the above analysis clarifies the upper and lower bounds of the stimulus velocity that supports rectified directional sensing (Fig. 8g,h). In the ultrasensitive regime (low *K*_I_), for stimulus within the time-window of rectification (V^*^_slow_<*V*_s_<V^*^_fast_), a large intracellular gradient of *R* is expected for the temporally increasing gradients (Fig. 8g; red curve) while it nearly vanishes for temporally decreasing gradients (Fig. 8g; cyan curve). In other words, a rectified sensing circuit implements low-pass filters with different cutoff times for rising and falling gradients (see also Supplementary Fig. 6; Supplementary Notes). At high *K*_I_ where rectified directional sensing becomes compromised (Fig. 7c,d; *K*_I_=0.1, Supplementary Fig. 5g–i), the intracellular gradient of *R* (the ratio *R*_+_/*R*_−_) always takes similar values in the rising and falling gradients (Fig. 8h). The time dependence is consistent with an earlier observation of cell movement in slowly diminishing gradients^34^ and may explain discrepancies between earlier works^32^,^33^.

## Discussion

The present study utilized precise and continuous displacement of a pulsatile gradient, thereby faithfully emulating the travelling wave stimulus of cAMP experienced in the aggregating field of cells. We demonstrated that cells are able to exhibit directional migration towards the incoming waves as observed *in vivo*. Cell migration in travelling waves of cAMP was indeed rectified, meaning that the cells migrated towards the incoming waves and did not reorient in the waveback. To the best of our knowledge, this is the first clear demonstration of *Dictyostelium* chemotaxis in artificially generated travelling waves of chemoattractant cAMP. Our observations indicate that no asymmetry in the gradient steepness between the wavefront and the waveback nor cell–cell contact is required for directional migration.

By generating various forms of dynamic gradients—inverted waves, transiently increasing or decreasing gradients, the current analysis demonstrated that Ras does not transduce gradient information when the mean concentration of cAMP is decreasing in the appropriate timescale. One of the key aspects of directional sensing in migrating cells is that it operates independent of cell motility^60^,^61^. Under a stationary gradient stimulation, Ras activation and PIP3 synthesis at the leading edge are observed even when cell motility is suppressed by Latrunculin^13^,^60^. Our present results clarified that this also applies to the ability of cells to filter out temporally decreasing gradients. Rectification is observed at the level of Ras activity, and this requires neither cell motility nor feedback from downstream PIP3 signalling.

The present results indicate that spatial sensing and temporal sensing can be understood under a unified framework. We have introduced the ultrasensitive LEGI model as a plausible and minimal extension of the basic LEGI framework and made use of its simplicity to analyse the basis of rectification. Our analysis suggests that rectification is separable from downstream amplification and/or an excitable circuit thus arises at or very close to the level of a LEGI-like circuitry. Biochemically, the rectifying property is expected to originate from regulation of Ras or its upstream signalling. Other variants of the LEGI model can also support rectification, however, these models require additional downstream signalling modules to realize the characteristic nonlinear transfer function (Supplementary Fig. 8; see Supplementary Note). For example, addition of the downstream PI3K-mediated amplification step (extended LEGI model Fig. 3b in ref. 52, amplified-LEGI^53^) to the basic LEGI model can provide necessary nonlinearity to support rectification (Supplementary Fig. 8e–l). However, the present analysis of LY-treated cells indicate that PI3K is required for overall amplification of Ras signal, but not for rectification (Fig. 4a,b; Supplementary Fig. 3a–f). While it is possible that Ras itself constitutes the amplifying/rectifying module downstream of an unidentified LEGI module close to the G-protein coupled receptor, the responses at the level of the heterotrimeric G-protein observed so far have been non-adaptive^19^ thus not LEGI-like in their property.

The other plausible source of nonlinearity is the feedback from F-actin that supports excitability^25^,^26^,^51^. In latrunculin-treated cells, RBD translocation was again rectified meaning that it occurred in temporally increasing gradients, not in decreasing gradients. Note, however, that RBD localization appeared more graded in space (Fig. 4c,d; Supplementary Fig. 3g–j) suggesting that nonlinearity that enhances the difference between the leading and trailing end of the cells has a separate origin than the rectification. In the light of the LEGI scheme, these observations suggest that either sequestering of the activator or diffusion of the inhibitor is F-actin dependent (Supplementary Fig. 7d–f). This is plausible considering that a membrane scaffold that associates with RasGEFs is known to translocate to the plasma membrane in a F-actin-dependent manner^17^. Finally, we should note that the system must operate near the point of inflection in the *Q*–*R* curve. This requires fine-tuning of the stationary state *Q*_0_ (Supplementary Fig. 5e), which also determines the pulse form of *R* and the degree of imperfectness of adaptation. Future works should address how such robustness is achieved in relation to complexities of the signalling network that were omitted in the current model.

In *Dictyostelum*, the rectified directional sensing together with the self-generated gradients enable long-distance cell migration by circumventing dissipation of the guidance cue and recycled use of spatial gradients. In sperm, the gradient perceived by the cell becomes a periodic stream of chemoattractant due to looping cell motion, thus in essence utilizes dynamic sensing^62^. Directional migration in neutrophils also appears to involve spatio-temporal mechanisms^40^,^63^,^64^. A similar rectified sensing may allow the immune cells to ignore subsiding signals from the site of wound infliction and inflammation. Although whether periodic travelling waves exist in migrating systems besides *Dictyostelium* remains an open question, neutrophil aggregation to the wound site is mediated by self-amplified signals^4^. The signals perceived by the cells in such developing fields of attraction are likely to be complex in their temporal patterns. The present insights on spatio-temporal sensing and rectification should be useful for the analysis of these and other cellular sensing.

## Methods

### DNA construct and cell strains

An expression vector for RFP tagged with the RBD of human Raf1 protein was based on GFP-RBD^13^,^24^. RFPmars^65^ was PCR amplified using primers 5′- AGATCTATGGCATCATCAGAAGATGTTATT -3′ (BglII-RFP) and 5′- GAATTCGATCCTGCACCTGTTGAATGTCTA -3′ (RFPΔTAA-EcoRI) using pHygRFPmars^25^ as a template. The GFP-RBD expression vector was replaced with RFPmars, purified and sequenced. The vector confers resistance to G418 and expression of RFP-RBD under a strong promoter. AX4 cells were transformed by electroporation and cloned following a standard protocol. To obtain cells co-expressing RFP and a Foster resonance energy transfer (FRET) sensor for cAMP Epac1-camps^66^, AX4 cells expressing Epac1-camps^43^ were transformed with pHygRFPmars^25^ and selected for G418 and hygromycin resistance. All transformants were cloned for further analysis.

### Cell preparation

For time-lapse imaging analysis of cell aggregation, the laboratory wild-type strain Ax4 of *D. discoideum* cells expressing the cAMP sensor Epac1-camps^43^ were employed. For cell tracking, cells co-expressing RFP and Epac1-camps were employed. Cells were grown axenically in modified HL-5 medium shaken at 22 °C with appropriate selection (10 μg ml^−1^ G418 and 60 μg ml^−1^ hygromycin). Growing cells were washed twice and resuspended in phosphate buffer (PB) (20 mM KH_2_PO_4_, 20 mM Na_2_HPO_4_, pH 6.5) at 6.5 × 10^6^ cells ml^−1^. For simultaneous observations of the cAMP relay and cell migration, Epac1-camps-expressing cells were mixed with RFP co-expressing cells in a 98:2 ratio. To obtain aggregating cell populations, 3 ml of cell suspension was deposited on the 1% agar plate (3 ml 1% agarose (BactoAgar, Difco) in PB; 60 mm dish (Iwaki)), and the supernatant was removed after 15 min. The plates were dried for additional 10 min in a sterile hood, and incubated at 22 °C for 4–5 h prior to observations. For live-cell time-lapse imaging, the cell monolayer together with the supporting agar (~1 cm^2^ area) was cut out from the plate and carefully placed upside down on a glass-bottom dish (MatTek) for cell tracking in two dimensions.

For experiments in the microfluidic chamber, cells expressing RFP-RBD were grown axenically in the growth medium containing 10 μg ml^−1^ G418. Washed cells were resuspended in PB at a density of 5 × 10^6^ cells ml^−1^ and shaken at 22 °C. After 1 h, cells were pulsed at a final concentration of 50 nM cAMP (Sigma) every 6 min for 2–2.5 h (for the experiments in Fig. 7) or for 4–4.5 h (for other experiments). For observations, starved cells were collected and resuspended in PB at a cell density of 1 to 2 × 10^5^ cells ml^−1^. The suspended cells were transferred to a microfluidic chamber by manual pipetting. Cells were settled for 30 min before the flow was applied to the chamber.

### Dynamically changing gradient stimulus

For precise control of concentration profiles of extracellular cAMP in space and time, we employed a microfluidics chamber (μ-slide 3-in-1; Ibidi) consisting of three inlets and a single outlet. Inlet and outlet channels were connected to a pneumatic pressure regulator (MFCS-FLEX; Fluigent) to monitor and precisely control the pressure and flow rates. The pressure control was automated by the MAESFLO software (Fluigent) using custom-made scripts that set the flow rates from the individual inlet dynamically over time. The rate of total flow from the three inlets was maintained at 33 μl min^−1^. Tubes from the buffer source with or without cAMP (Sigma; molecular weight=329) were connected to the inlets in the configuration shown in the schematics (Figs 2a and 3a,e,i,m). As a marker for the stimulus profile, fluorescein (Wako; molecular weight=332) at a final concentration of 3 μM was included in the stimulus solution. A concentration of 1 μM cAMP was chosen as the stimulus source for all experiments, except for the inverse travelling wave experiments (Fig. 5i–l; Supplementary Fig. 3a–c,g,h) where 10 nM cAMP was also used. For elevated basal cAMP, the buffer-only pools were replaced with either 1 or 10 nM cAMP.

For spatially uniform stimuli, the left and right inlets were connected to a pair of syringe pumps (NE-1002X; New Era Pump Systems Inc., NY), and the centre inlet was sealed with a plug. The total flow rate of the solutions was either 30 μl min^−1^ (Fig. 7) or 120 μl min^−1^ (Supplementary Fig. 4). Under the present conditions, the estimated shear stress^67^,^68^ experienced by the cell was <0.03 Pa. This is more than one order of magnitude smaller than the shear force required to induce migration (>0.5 Pa)^67^ or to detach the cells (>1 Pa)^69^.

### Live-cell imaging

Image data were obtained using an inverted microscope (IX-81; Olympus) equipped with a confocal multibeam scanning unit (CSU-X1; Yokogawa) and an EM-CCD camera (Evolve 512; Photometrics). To detect fluorescein and RFP fluorescence, a bandpass filter (510–550 nm; BA510-550, Olympus) and a broad-spectrum filter (>575 nm; BA575IF, Olympus) were used, respectively. For FRET-based cAMP measurements, an excitation filter (BP425-445HQ, Olympus) and a dichroic mirror (DM450, Olympus) were used. Bandpass filters (BA460-510HQ, Olympus; BA515-560HQ, Olympus) were used for cyan fluorescent protein and yellow fluorescent protein fluorescence, respectively. Objective lens used were × 4 (UplanSApo, numerical aperture (NA) 0.16) for the quantification of the stimulus profiles, × 20 oil immersion (UplanSApo, NA 0.85) and × 60 oil immersion (PlanApo N, NA 1.42) for cell migration and FRET measurements, × 20 oil immersion for cell migration in the microfluidics chamber and × 60 oil immersion for simultaneous measurements of RBD translocation and cell migration in the microfluidics chamber. Some of the cell migration data were acquired with a single-beam scanning confocal microscope (Nikon A1R) with a × 100 oil immersion objective lens (PlanApo λ, NA 1.45). Fluorescence images were acquired at 1–30-s intervals and stored as Tagged Image File Format (TIFF) files. Obtained data were later analysed using ImageJ and MATLAB (MathWorks). All live-cell imaging was performed at 22 °C.

### Image processing

Cell tracking and fluorescence signal quantification were performed with custom programs written in ImageJ and MATLAB (MathWorks). To acquire changes in the FRET efficiency from Epac1-camps-expressing cells, the ratio of the fluorescence intensities in the cyan fluorescent protein (*I*_485_) and yellow fluorescent protein (*I*_540_) channels were averaged over a 40-μm square region around the centre of a cell at each time point. The mean ratio (*I*_485_/*I*_540_) was further averaged at each phase of an oscillation period then normalized by subtracting linear trends of the signal (normalized *I*_485_/*I*_540_).

For quantification of RBD translocation to the cell membrane, a 1-μm-wide region inside the cell outline and the cytosolic region were defined by binarization. For dynamically changing gradients, translocation of RFP-RBD to the cell membrane was quantified by taking the maximum fluorescence intensity in the membrane region in the direction *θ* (0<*θ*<2*π)* from the center; *I*_mem_(*θ*, *t*). The angle *θ*=*π*/2 faces the positive direction (right-hand side of the chamber). As an indicator of RBD localization, the averaged intensity of the membrane-cytosolic ratio was weighted by the alignment with the wave direction by computing, 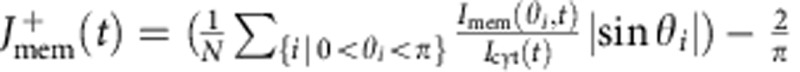, for the positive half of a cell periphery, and 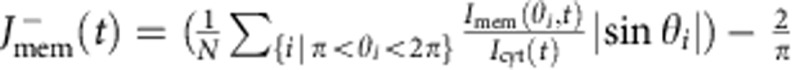, for the remaining half. Here, the discretized angle 
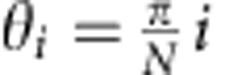
 for 1≤*i*≤2*N* where *N*=90. For normalization, the mean intensity of the cytosolic fluorescence, *I*_cyt_ (*t*) was used. We occasionally encountered extremely polarized cells with marked movement in the *z* axis direction. These cells were excluded from the present analysis due to difficulty in accurate tracking of the RBD translocation.

For quantification of the spatial and temporal changes in the cAMP levels, bias due to non-uniform illumination was removed following the flat-field correction method^70^. In brief, the spatial and temporal profiles of cAMP concentrations *I*_cAMP_ (*x*, *y, t*) were obtained by calculating 
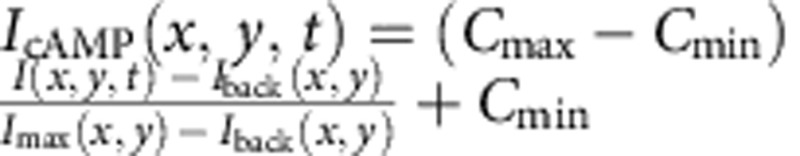
. Here, *I*(*x*, *y, t*) was fluorescence profile of the fluorescein indicator obtained during the stimulus experiments. The intensity profiles of the maximum *I*_max_(*x*, *y*) and the background *I*_back_(*x*, *y*) were obtained by capturing fluorescence of uniform concentration fields of the cAMP solution with or without fluorescein, respectively. *C*_max_ and *C*_min_ were the maximum and minimum concentrations of cAMP at the source, respectively. The calibration data were obtained prior to stimulus experiments per chamber on a daily basis.

To estimate the duration of the stimulus (that is, wave passage time), profiles of the wave stimulus were fitted by a Gaussian curve moving at a constant speed *V*_S_, *S*(*x,t*)=*B*_1_exp[−(*x*+*V*_S_*t*−*x*_0_)^2^/2*σ*^2^]+*B*_2_, using the nonlinear least-squares method. The wave passage time was defined by the time-window during which the stimulus intensities were above 0.01% of the peak intensity at a position *x*, that is *S*(*x,t*)>10^−4^ *B*_1_+*B*_2_. Estimation of the pulse width followed the same criterion (>0.01% of the peak intensity).

Quantification of Ras activation to uniform stimulus was performed as follows. The mean fluorescence intensities from the cytosolic region of cells under mock stimulation (no cAMP) *I*_cyt, 0_(*t*) was obtained separately to remove the effect of photobleaching. To obtain the standardized cytosolic RFP-RBD fluorescence, we calculated 
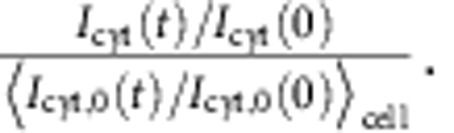
 Here, ‹*›_cell_ is an ensemble average of cells. The linear trend was further subtracted and normalized to obtain *J*_cyt_(*t*) that takes the value of 1 at *t*=0 and *t*=*t*_end_ (the end point of the time-lapse recording). Fluorescent intensities of the membrane-bound RFP-RBD prior to spatially uniform stimulation, *I*_mem, uniform_(0), were quantified as follows. The entire membrane region of a cell was divided into 16 segments. The pixel intensity was ranked, and the top 20–40th percentiles were averaged per segment to remove noise. This was further averaged over all segments to yield *I*_mem, uniform_(0). As an indicator of Ras activity, we computed 
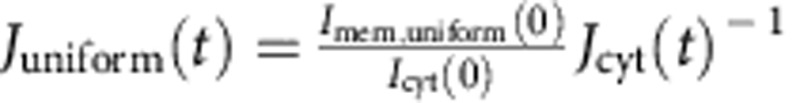
.

### Mathematical modelling

In the basic LEGI formulation^8^, *F*(*R*)=*k*_A_(*R*_tot_−*R*) and *G*(*R*)=*k*_I_*R* are employed, where *R*_tot_−*R* and *R* are concentrations of ‘*R’* in the inactive and the active form, respectively. As a biochemically natural extension of the LEGI scheme, we adopted the Michaelis–Menten form in the regulation of *R*, such that




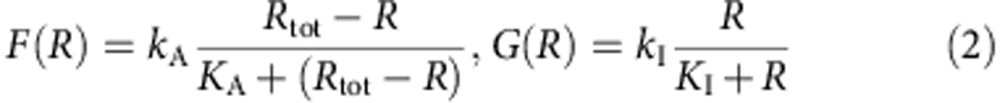




This constitutes the so-called push-pull type reaction where two antagonistic enzymes reversibly modify the substrate *R*. In this paper, we refer to this modified form (equations (1) and (2)) as the ‘ultrasensitive LEGI model’. Note that when the substrate is limited, that is, *K*_A_/*R*_tot_ and *K*_I_/*R*_tot_ are large, *F*(*R*)~*k*_A_(*R*_tot_−*R*) and *G*(*R*)~*k*_I_*R* so that equation (1) recovers the basic LEGI equation (first-order kinetics). For the simulations of directional sensing in various concentration fields, the response *A*, *I* and *R* at the cell ends were considered in one-dimensional space along the gradient (Fig. 6a). Under these assumptions, we obtain the following equations:




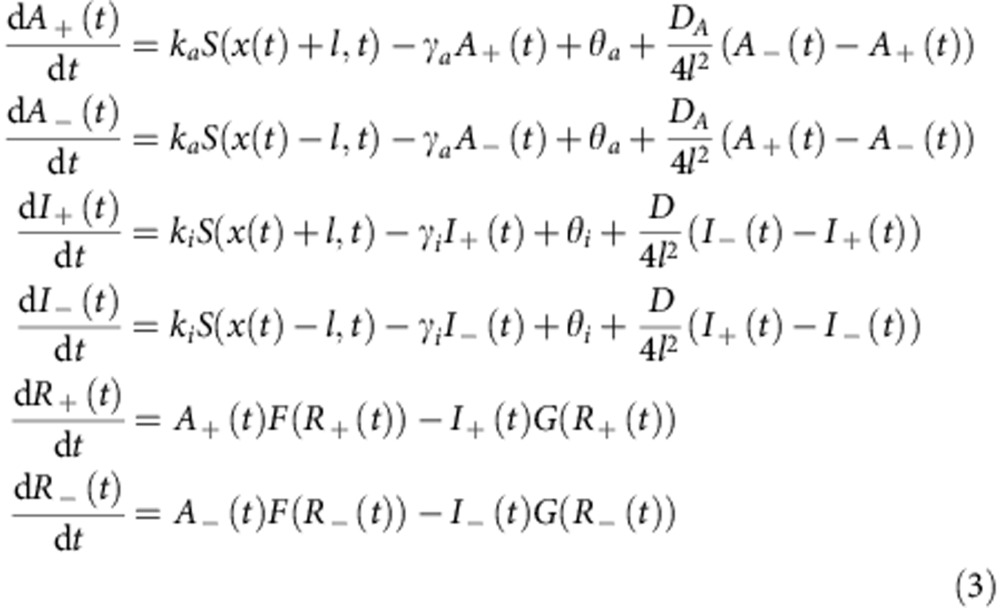




*A*_+_, *A*_−_, *I*_+_, *I*_−_, *R*_+_ and *R*_−_ indicate the variables *A*, *I* and *R* at the respective ends of a cell of length *2l* positioned at *x*=*x*(*t*). The subscript ‘+’ means variables at the positive end of a cell (*x*(*t*)+*l*) and ‘−’ at the negative end (*x*(*t*)−*l*). Parameter *D*_A_ and *D* (*D*_A_≪*D*) determine diffusibility of the activator ‘*A*’ and the inhibitor *‘I’* inside the cell, respectively. According to the LEGI scheme, we assumed the inhibitor ‘*I’* to diffuse rapidly and act globally while the activator ‘*A*’ to act locally. We chose *D*_A_=0 unless otherwise noted. In numerical calculations, time *t* and space *x* take the absolute physical units (s and μm). *A, I* and *R* are normalized to their respective a.u. The cell length is fixed at 2*l*=15 μm. Unless otherwise noted, we study conditions where cell migration speed is sufficiently small compared with the speed of the travelling wave, so that the cell position is fixed at *x*(*t*)=0. For gradient stimulation, the following signal profiles were adopted:

(i) Travelling waves (Figs 6b,d,e and 7c,e; Supplementary Figs 6 and 8d,h,l,p).

Travelling wave experiments (Figs 2, 4, 5a–h and 7g–j) were simulated by employing a Gaussian profile moving at a constant speed *V*_S_; *S*(*x,t*)=exp[−*κ*(*x*+*V*_S_*t*)^2^]. For the sake of comparison with the experiments, the pulse width of the stimulus *L*_P_ was divided by the wave transit time to obtain the wave velocity *V*_S_. We chose *κ*=6.25 × 10^−5^ μm^−1^ and *L*_P_=800 μm to closely match the current experimental values (Supplementary Fig. 1; also see the main text).

(ii) Temporally changing gradients (Fig. 6f–i; Fig. 8).

To simulate temporally increasing gradients (Fig. 3a–d), we set *S*(*x,t*)=(1+tanh[*b*(*x*+*V*_S_*t*)])/2. Here, *b*=0.015 μm^−1^ is chosen to obtain the same maximal steepness of the gradient as in (i). Similarly, for temporally decreasing gradients (Fig. 3e–h), we set *S*(*x,t*)=(1+tanh[*b*(*x*−*V*_S_*t*)])/2. For the simulation at Fig. 6f–i, to imitate the profiles in the experiments, the wave velocity *V*_S_=180 μm min^−1^ was chosen.

(iii) Inverse travelling waves (Fig. 6j,k).

To simulate inverse travelling waves (Figs 3i–l and 5i–l; Supplementary Fig. 3), a ‘reverse Gaussian’ signal *S*(*x,t*)=1−exp[−*κ*(*x*+*V*_S_*t*)^2^] was employed. The parameter values were the same as those in the Gaussian signal (i).

(iv) Alternating gradients (Fig. 6l,m).

To simulate the alternating gradients (Fig. 3m–p), we imposed *S*(*x,t*)=1+(tanh[*b*(*xl*_0_ +*l*_0_*M*(*t/τ*))]−tanh[*b*(*x*+*l*_0_+*l*_0_*M*(*t/τ*))])/2 where *M*(*t*) is the triangle-wave function. Parameter values are *b*=0.015 μm^−1^, *l*_0_=350 μm and *τ*=6.0 min. The important difference from the inverse wave stimulus is that the signal intensity oscillates at a period half of that of the spatial gradient (Figs 3i,m and 6j,l).

## Author contributions

A.N. and S.S. designed the research. A.N. and D.I. performed experiments and analysed the data. A.N., S.I. and S.S. designed and analysed the model. S.I. carried out theoretical analysis and simulations. S.S. oversaw and coordinated data collection and analysis and contributed to all aspects of data interpretation. A.N., S.I. and S.S. wrote the paper.

## Additional information

**How to cite this article:** Nakajima, A. *et al*. Rectified directional sensing in long-range cell migration. *Nat. Commun.* 5:5367 doi: 10.1038/ncomms6367 (2014).

## Supplementary Material

Supplementary Information Supplementary Figures 1-8, Supplementary Tables 1, Supplementary Notes and Supplementary References

Supplementary Movie 1 - Propagating cAMP waves and cell migration in aggregating Dictyostelium cells Simultaneous observation of cAMP waves and cell migration in mixed population of cells expressing a cAMP sensor Epac1-camps (98% of the total cells) and cells co-expressing RFP (2% of the total cells). (Left panel) Propagating cAMP waves quantified by the ratio of the signal intensities (I485/I540) from the Epac1-camps probe (high concentration of cAMP, yellow; low concentration, purple). (Middle panel) Frame-subtracted ratio images (20 sec interval) ( d (I485/I540)/dt > 0, green; d (I485/I540)/dt ≤ 0, black). (Right panel) Cell migration in a cell aggregate. RFP signals (magenta) are superimposed on top of bright field transmitted light images (grayscale). Images were acquired every 20 seconds for total duration of 150 min. Time from nutrient removal is indicated at the bottom right corner. The field of view is 0.4 mm × 1.2 mm.

Supplementary Movie 2 - Directional cell migration induced by artificially generated cAMP wave stimuli. Overlaid images of the imposed wave profile (green) and the migrating cells (magenta). The period of wave stimuli was 6.5 min. Images were acquired every 15 seconds, and the duration of the movie is 26 min. The field of view is 0.3 mm × 0.3 mm.

Supplementary Movie 3 - Ras activation induced by the wave stimulus. Images of RFP-RBD translocation of a migrating cell (magenta) in a traveling wave stimulus (green). Fluorescence intensity in the green channel is shown in a logarithmic scale for visualization. Images were acquired every 15 seconds, and the duration of the movie is 10.5 min. The field of view is 0.06 mm × 0.06 mm.
