# Supplementary material for: Rectified directional sensing in long-range cell migration
Source: Nat Commun. 2014 Nov 6;5:5367. doi: 10.1038/ncomms6367 (PMC4272253; doi:10.1038/ncomms6367)
Supplement: Supplementary Information — Supplementary Figures 1-8, Supplementary Tables 1, Supplementary Notes and Supplementary References [file ncomms6367-s1.pdf]

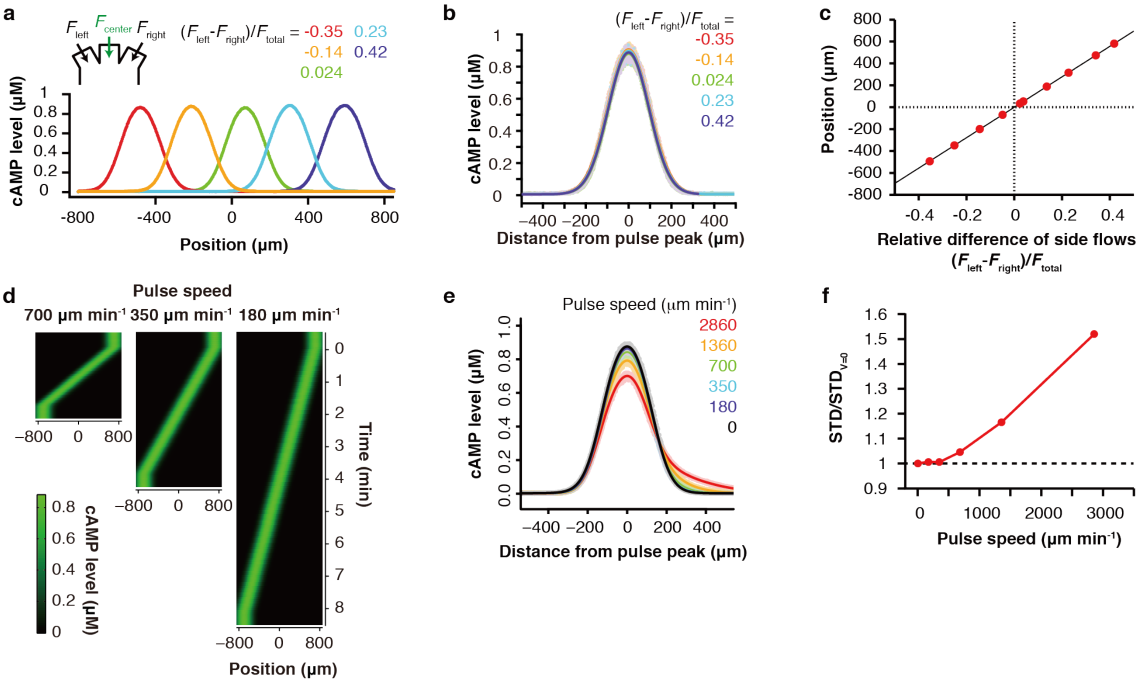

Supplementary Figure 1. Generation of spatially symmetric traveling waves. (**a**) Infusing flows and the positioning of a stationary standing wave. The relative differences between the side flows shifts the pulse position; from left to right $(F_{\text{left}} - F_{\text{right}})/F_{\text{total}}$= -0.35 (red), -0.14 (yellow), 0.024 (green), 0.23 (cyan), and 0.42 (purple). (**b**) Standing waves at different locations showed near perfect overlaps in their spatial profiles. The shaded region indicates the size of temporal fluctuations. Data were acquired at 2 sec intervals for 1 min. Standing waves were fitted by a Gaussian curve using non-linear mean square methods (solid lines) and their peak positions were used as the center for alignment. (**c**) Relative difference in the side flows and the pulse positions. The black line indicates a least square fitting. (**d**-**f**) Linear ramping of the relative flow rate and the movement of the pulse position. The total flow rate was fixed at 33 μL min^{-1}. Kymographs of traveling waves propagating at 700 μm min^{-1} (left), 350 μm min^{-1} (middle), and 180 μm min^{-1} (right) (d). Spatial profiles were conserved for propagation speed < 1000 μm min^{-1} (e). The direction of wave propagation was in the negative direction in the *x*-axis (from right to left in e). The profiles were obtained by non-parametric regression using smoothing splines (solid lines). Pale colors show temporal fluctuations during wave propagation. (**f**) Standard deviation of the time averaged spatial pulse profile at different propagation speed relative to the stationary standing wave (pulse speed = 0 μm min^{-1}, black line in (e)). Profiles of waves at a lower speed (< 500 μm min^{-1}) show minimal deviation from those of standing waves.

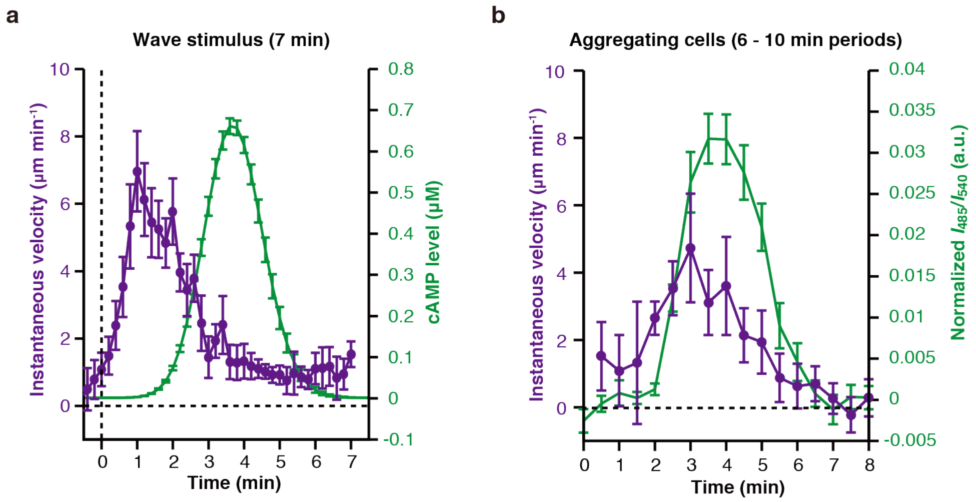

Supplementary Figure 2. Artificial traveling waves closely emulate the cell movement in the aggregation field. (**a**) Changes in the average instantaneous cell velocity (purple; n = 21) in relation to applied cAMP traveling waves (green; fluorescein). Time t = 0 indicated the point at which the stimulus concentration exceeded a threshold (0.01% of maximum concentration) (see Methods). (**b**) Instantaneous velocity (purple) and the cytosolic cAMP level (green; Epac1camps) in aggregating cells. Data were taken from cells (n = 7; see Methods) in the early aggregation stage when the period of cAMP oscillations were 6 – 10 min. Error bars indicate s.e.m.

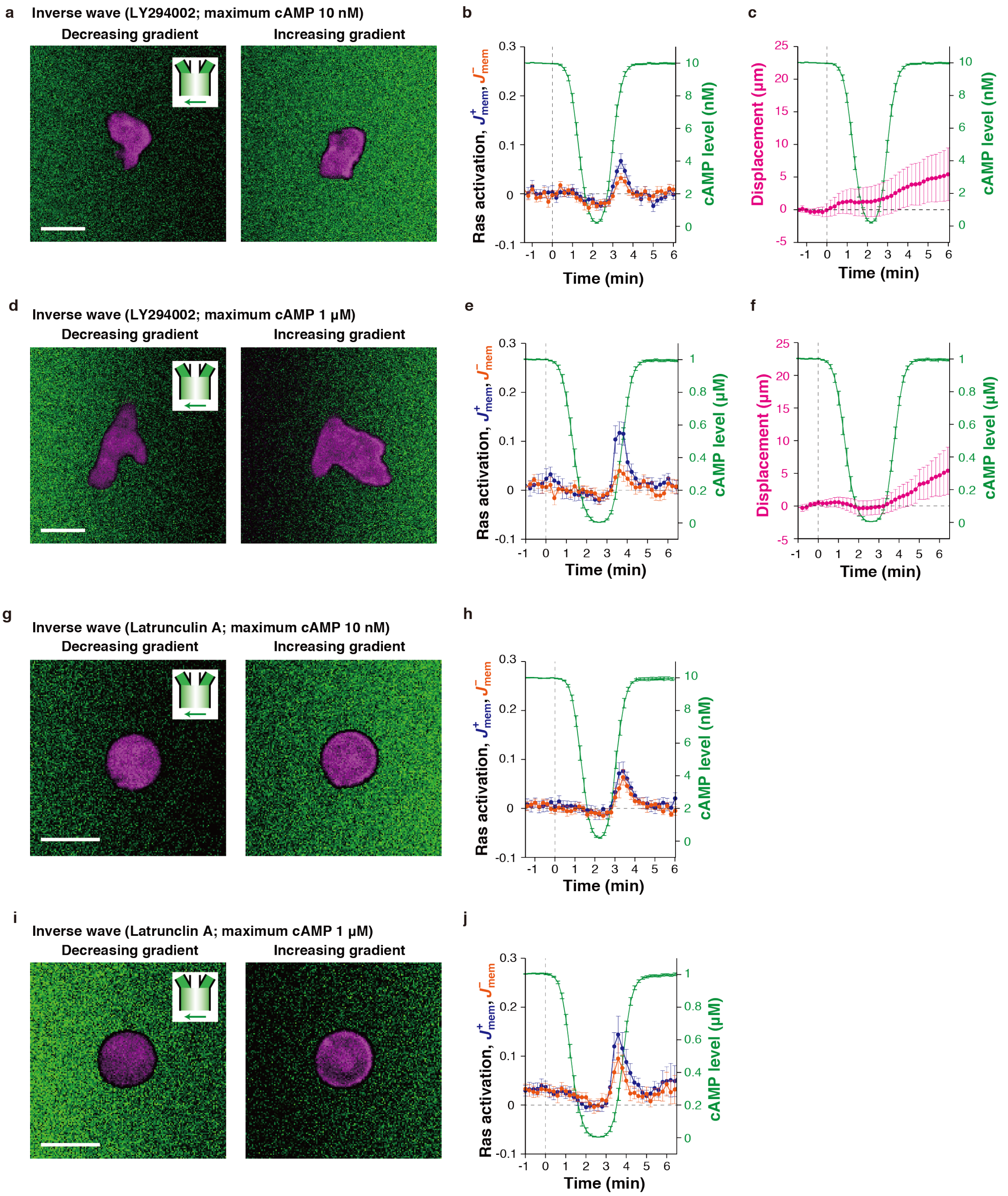

Supplementary Figure 3. Pharmacologically treated cells show rectification in the inverted wave stimulus. (**a-f**) Cells treated with 50 μM LY294002 (a-f) and 5 μM Latrunculin A (g-j). The maximum concentrations was 10 nM (a-c, g, and h) and 1 μM (d-f, i, and j). Representative images of localized Ras activation (RFP-RBD; magenta) and cAMP gradients (fluorescein; green) (a, d, g, and i). Time series of RBD translocation to the positive side (J^{+}_{mem}, blue) and the negative side (J^{-}_{mem}, orange) (b, e, h, and j). Time series of cell displacement (c and f; magenta). Extracellular cAMP levels (b, c, e, f, h, and j; green). Time

t = 0 indicates the point at which the stimulus concentration was below a threshold (99.5% of the maximum). Data plots are averages ± s.e.m. (error bars) over n = 23 (b and c), 13 (e and f), 12 (h) and 13 (j) cells. Scale bar, 10 μm (a, d, g, and i).

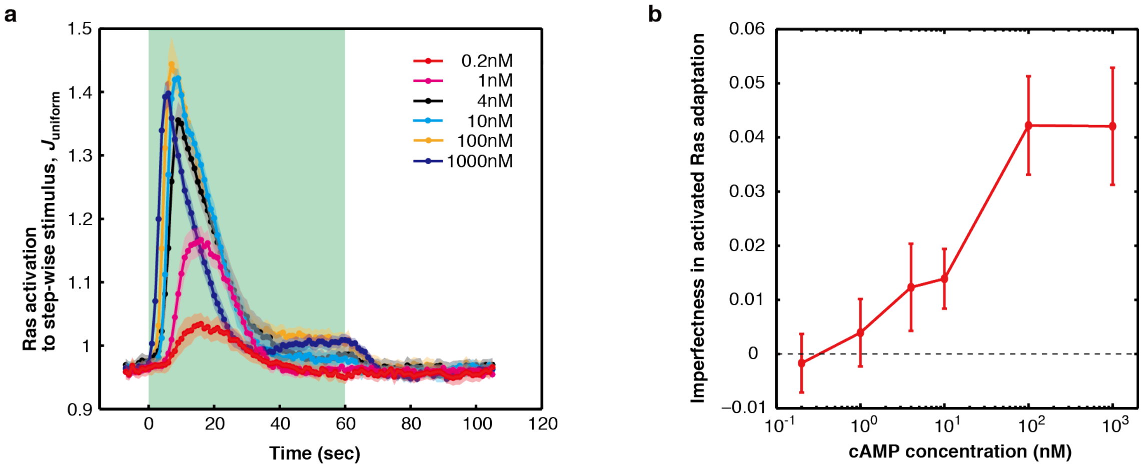

Supplementary Figure 4. The adaptive Ras response was rectified in a wide range of spatially-uniform changes in the cAMP concentrations. (**a**) Transient changes in the Ras activity to spatially-uniform increase and decrease of cAMP concentrations in fully aggregation-competent cells (4.5 h pulsing with cAMP). The concentration of cAMP was increased from 0M to the concentrations indicated for 60 seconds (green shaded area) followed by a decrease to 0M. The membrane translocation of RBD were quantified and averaged over n = 16 (0.2 nM), 15 (1 nM), 18 (4 nM), 17 (10 nM), 14 (100 nM), and 18 (1000 nM) cells. Error bars indicate s.e.m. (**b**) Imperfectness of adaptation in the Ras response. The average difference in the level of RBD translocation before (t = -10 to -5 sec) and after prolonged stimulus exposure (t = 55 to 60 sec). The dashed line indicates a perfect adapting case. Error bars indicate s.e.m.

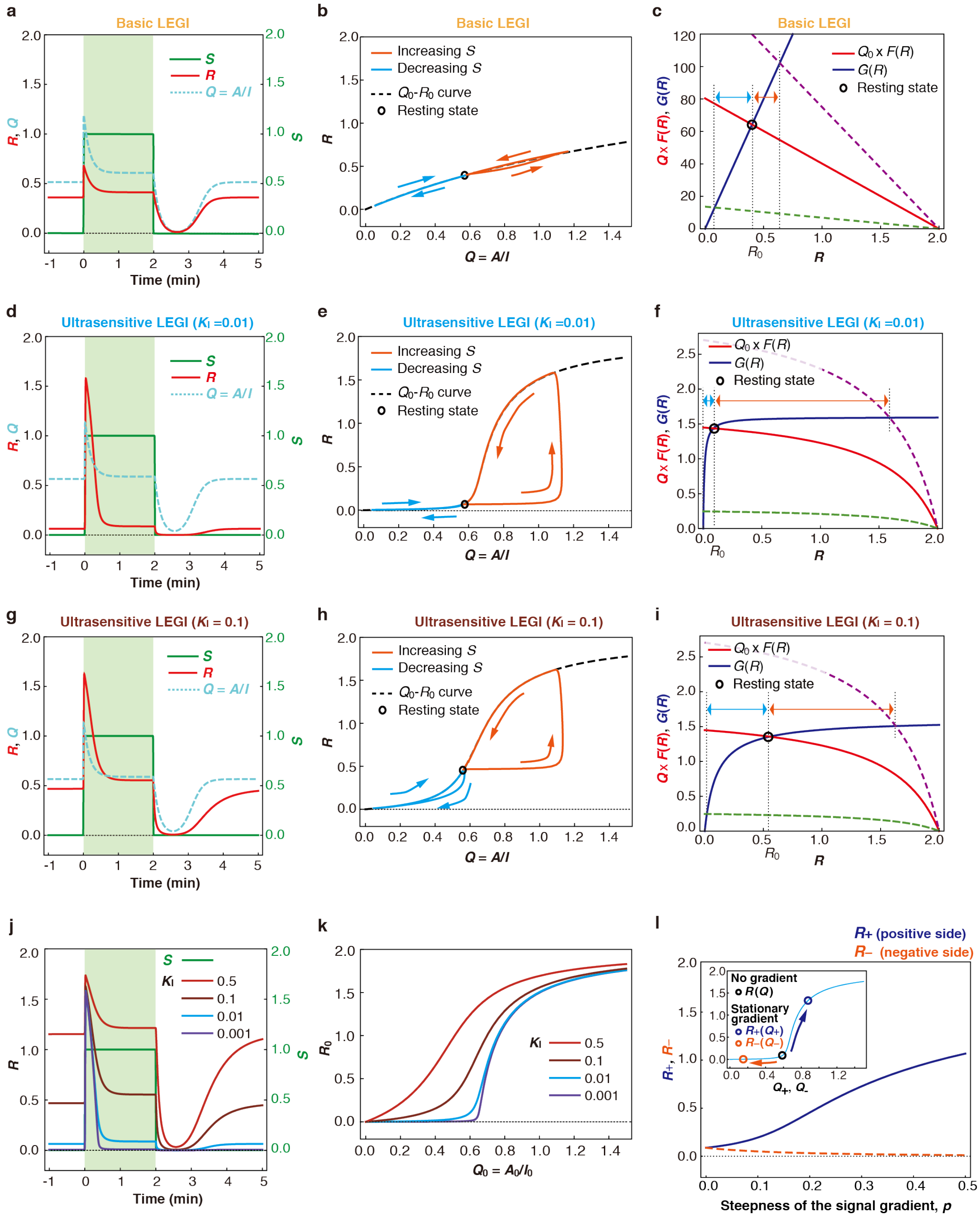

Supplementary Figure 5. The rectified response arises from asymmetry in the transfer function. (**a-i**) Adaptive response to spatially-uniform stimuli in the basic LEGI model (a-c), and the ultrasensitive LEGI model for $K_I = 0.01$ (d-f) and $K_I = 0.1$ (g-i). Response in Q (cyan) and R (red) to uniform stimuli (green) (a, d, and g). Response profiles to spatially-uniform stimuli in the Q-R plane (b, e, and h). Relation between Q and R in the stationary state (black dotted line). Activation and deactivation rates of R (c, f, and i). The intersect of $Q_0 \times F(R)$ and

$G(R)$ represents the resting state of R. The transient changes in $Q \times F(R)$ in response to a step-wise increase and decrease in S (broken lines; purple for step increase (Q = 1.1); green for step decrease (Q = 0.1)). (**j**) K_I dependence of the response R to uniform stimuli (green). **(k)** Stationary state $R = R_0$ plotted against $Q = Q_0$. Strong ultrasensitivity appears for small K_I. (**l**) Stationary states of R in the ultrasensitive LEGI at $K_I = 0.01$ in gradients $S(x) = S_0(1+px/l)$ for $S_0 = 0.5$ (R_+, blue, and R_-, orange). Stationary states for steepness $p = 0.8$ (R_+, blue; R_-, orange) or no gradient (R, black) in the Q-R plane (l, inset). For model parameters, see Supplementary Table 1.

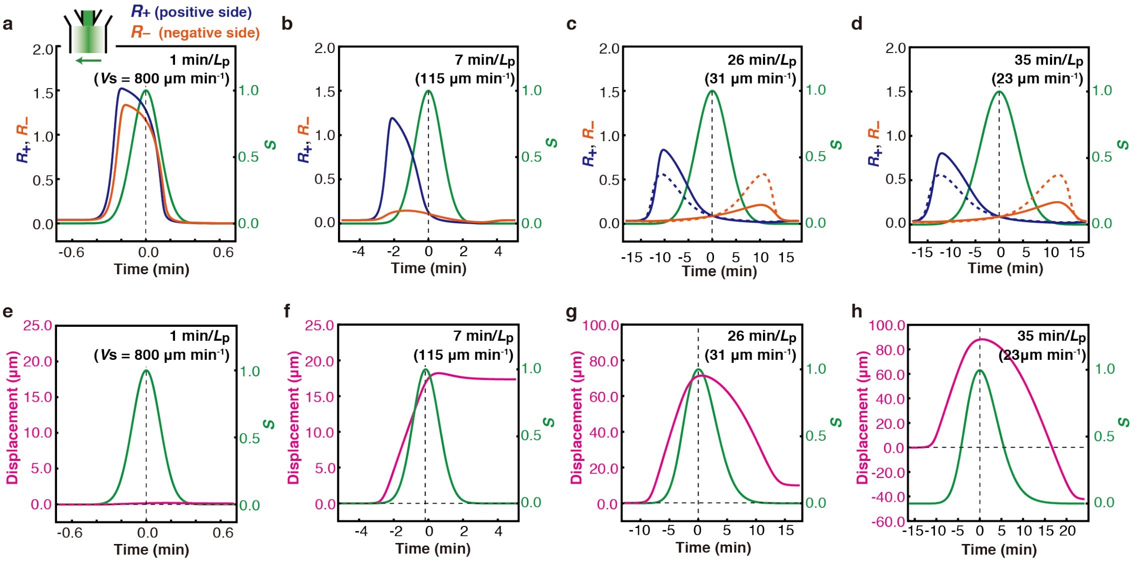

Supplementary Figure 6. Rectified response in the ultrasensitive LEGI model explains the time dependence of cell migration and its direction in the wave stimulus. **(a-d)** Response of R_+ (blue) and R_- (orange) in traveling wave stimulus (green) (K_I = 0.01). The wave transit time was 1 min/L_P (a), 7 min/L_P (b), 26 min/L_P (c), and 35 min/L_P (d). **(e-h)** Simulations of cell displacement (magenta) (Supplementary equation (1) and (2)) coupled to the ultrasensitive LEGI model for traveling wave stimulus of 1 min/L_P (e), 7 min/L_P (f), 26 min/L_P (g), and 35 min/L_P (h) duration. The temporal profile of the signal S at the cell position (e-h, green). For model parameters, see Supplementary Table 1.

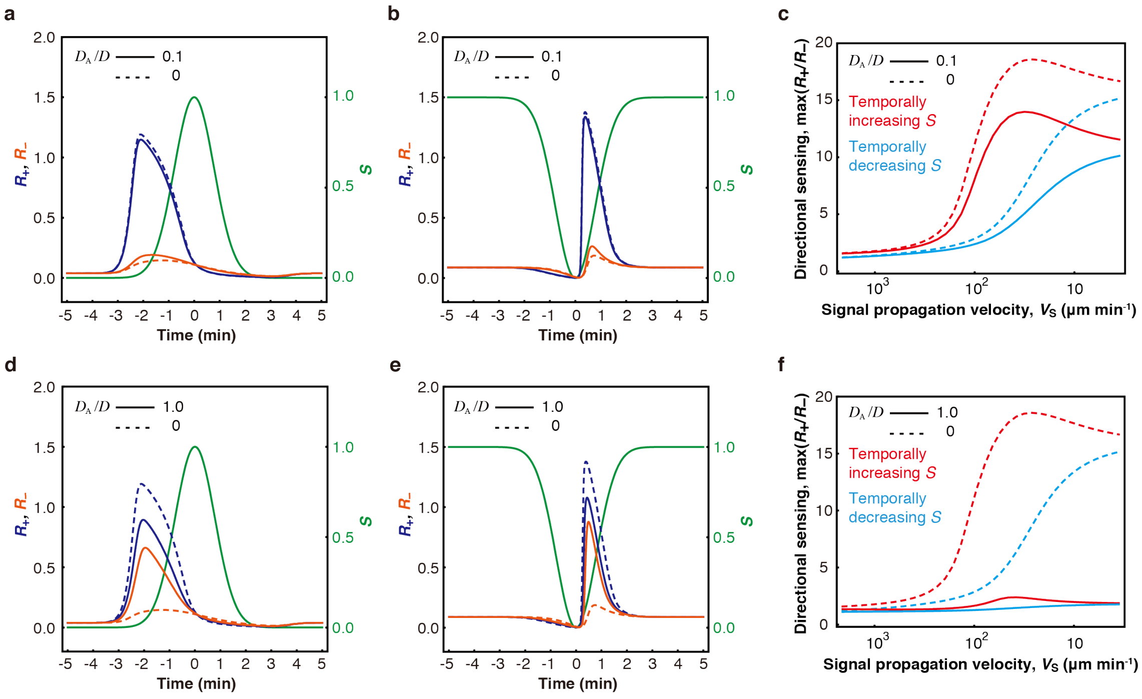

Supplementary Figure 7. Numerical simulations with activator diffusion. (**a-c**) Rectified directional sensing is preserved in the presence of activator diffusion (D_A = 3μm^2 sec^{-1}, D = 30 μm^2 sec^{-1}). Activation of R in response to traveling wave (a) or inverse traveling wave (b) stimulus. The maximum of the ratio R_+/R_- in response to temporally increasing (red) and decreasing (cyan) spatial gradients (c). Results without activator diffusion (D_A = 0; Fig. 6e and k, Fig. 8g) are shown in dotted lines for comparison. Note that the time-window of signal duration required for rectified directional sensing does not largely deviate from the case without activator diffusion. (**d-f**) Numerical simulations for $D_A = D$ (= 30 μm^2 sec^{-1}). The difference in the output R between positive and negative ends (R_+ and R_-) becomes small (d, e) similar to the profile of Ras activation in latrunculin-treatead cells. Maximum values of the ratio R_+/R_- is small indicating very weak directional sensing (f). For model parameters, see Supplementary Table 1.

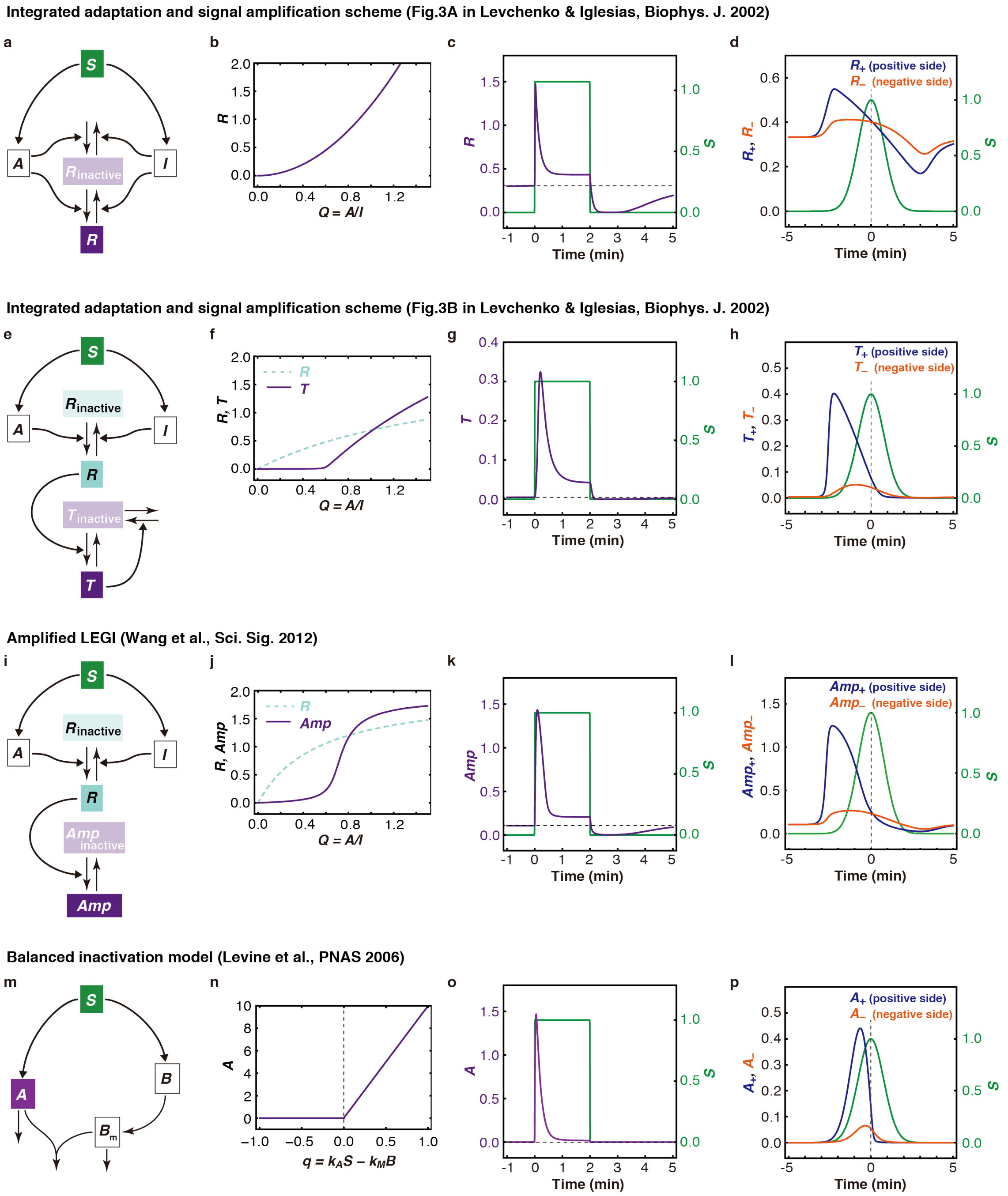

Supplementary Figure 8. Rectification in other LEGI-related models. (**a-d**) Levchenko-Iglesias Model A (Fig. 3A in Supplementary Ref 2). (**e-h**) Levchenko-Iglesias Model B (Fig. 3B in Supplementary Ref 2). (**i-l**) Amplified LEGI model[1]. **(m-p)** Balanced inactivation model[2]. Schematics of earlier models (a, e, i, and m), characteristic response curves (b, f, j, and n), response to spatially-uniform stimulus (c, g, k, and o), and response to traveling wave stimulus (d, h, l, and p). Parameter values were chosen so as to realize the nonlinear transfer function. (b, f, j, n; see Supplementary Notes).

Supplementary Table 1. Model Parameters used in the study.

Parameter	Value*
$\boldsymbol{k}_{\mathbf{a}}$	3.3 sec^{-1}
$\boldsymbol{k}_{\mathbf{i}}$	2.8 sec^{-1}
$\boldsymbol{\gamma}_{\mathbf{a}}$	0.2 sec^{-1}
$\boldsymbol{\gamma}_{\mathbf{i}}$	0.1 sec^{-1}
$\boldsymbol{\theta}_{\mathbf{a}}$	1.0×10^{-3} sec^{-1}
$\boldsymbol{\theta}_{\mathbf{i}}$	1.0×10^{-3} sec^{-1}
$\boldsymbol{R}_{\mathbf{tot}}$	2.0
$\boldsymbol{k}_{\mathbf{A}}$	68 sec^{-1} (basic LEGI), 3.0 sec^{-1} (ultrasensitive LEGI)
$\boldsymbol{k}_{\mathbf{I}}$	160 sec^{-1} (basic LEGI), 1.6 sec^{-1} (ultrasensitive LEGI)
$\boldsymbol{K}_{\mathbf{A}}$	0.44
$\boldsymbol{K}_{\mathbf{I}}$	specified in the text and figure captions
$\boldsymbol{l}$	7.5 μm
$\boldsymbol{D}$	30 μm^2 sec^{-1}
$\boldsymbol{D}_{\mathbf{A}}$	0 μm^2 sec^{-1} unless otherwise noted

* Concentrations of molecules are in an arbitrary unit.

Supplementary Notes

The basis of rectification in the ultrasensitive LEGI model

In order for the response R to adapt to spatially-uniform stimuli (equation (1)), the level of R at the steady state should not depend on the value of S. Here, adaptation refers to the recovery of the response to the pre-stimulus level under persistent stimulation (Supplementary Fig. 5a). Under constant and spatially-uniform stimuli $S(x,t) = S_0$, one obtains a fixed point $A = A_0 \stackrel{\text{def}}{=} \gamma_a^{-1}(k_a S_0 + \theta_a)$ and $I = I_0 \stackrel{\text{def}}{=} \gamma_i^{-1}(k_i S_0 + \theta_i)$. By substituting $dR/dt = 0$ in the third equation of equation (1), we see that the fixed point $R = R_0$ is determined by $Q_0 \stackrel{\text{def}}{=} A_0/I_0$ that satisfies $F(R_0)Q_0 = G(R_0)$. When the adaptation response to the sustained stimulus is nearly perfect, θ_a and θ_i should be negligibly small. In this case, we see that $Q_0 = \gamma_a^{-1}(k_a S_0 + \theta_a)/\gamma_i^{-1}(k_i S_0 + \theta_i) \approx \gamma_a^{-1}k_a/\gamma_i^{-1}k_i$. Thus Q_0 is independent of S_0, so is R_0. In general, it is assumed that $\gamma_i < \gamma_a$, so that the activator 'A' responds to the change in the stimulus 'S' faster than the inhibitor 'I', thereby R exhibits a transient positive (negative) response to the increase (decrease) of S. These features in the model form the basis of the response transient and adaptation.

In both the basic and the ultrasensitive LEGI model, the activator-inhibitor ratio $Q(t)$ ($=A(t)/I(t)$) transiently increase or decrease upon uniform increase or decrease in $S(t)$ respectively (Supplementary Fig. 5a, d and g). In the basic LEGI model, the output $R(t)$ exhibits not only a positive response to stimulus increase but also a strong undershooting response, i.e. non-rectified adaptive response, to the stimulus decrease (Supplementary Fig. 5a). On the other hand, the output $R(t)$ shifts only positively in the ultrasensitive LEGI model for low K_I ($K_I = 0.01$) (Supplementary Fig. 5d and j). In the case of high K_I ($K_I = 0.1$), $R(t)$ exhibits a marked undershoot similar to the original LEGI model (Supplementary Fig. 5g and j). As described in the main text, the extended model (equation (1)-(3)) is ultrasensitive to the activator-inhibitor ratio (A/I) (Fig. 6c and Supplementary Fig. 5k). This is a natural outcome of the near-zero-order kinetics of the inhibitory reaction $G(R)$ that outcompetes the activation reaction $F(R)$ when K_I is small relative to R_{tot}[3]. As a consequence, the stationary value of R ($= R_0$) becomes insensitive to changes in the stationary value of Q ($= Q_0$) for small Q_0 (Supplementary Fig. 5k). This is in sharp contrast to the basic LEGI model where changes in Q_0 are proportionately transferred to the response in R_0 (Fig. 6c).

The asymmetric $R(t)$ response to changes in $Q(t)$ can be understood from the trajectories in the Q-R plane (Supplementary Fig. 5b, e, and h). The response $R(t)$ moves

along the Q_0-R_0 curve after an abrupt change in $Q(t)$ induced by the change in S. In the basic LEGI model, the transients in $R(t)$ from the resting state are almost symmetric in magnitude for increasing and decreasing $Q(t)$ (Supplementary Fig. 5b; orange for increasing and cyan for decreasing stimuli) due to linearity in its response function (Q_0-R_0 curve) (broken black line in Supplementary Fig. 5b). The symmetry is evident in the transient changes of $Q{\times}F(R)$ in response to S (Supplementary Fig. 5c). The resting state of R (R_0) is at the intersection point of two curves $Q_0{\times}F(R)$ and $G(R)$ from the relationship $Q_0F(R_0) = G(R_0)$. Because $F(R)$ and $G(R)$ are linear functions of R in the basic LEGI model, both increase (red to purple) and decrease (red to green) in $Q(t)$ result in equally large positive and negative shifts of the intersect which translates to a symmetric change in $R(t)$ (Supplementary Fig. 5c). In contrast, in the ultrasensitive LEGI model at low K_I ($K_I = 0.01$), the transients of $R(t)$ from the resting state are highly asymmetric with respect to the sign of time derivative of $Q(t)$ (Supplementary Fig. 5e). This is easily understood from the ultrasensitvity in the response function, which translates increase in $Q(t)$ to a large increase in $R(t)$, but filters out decrease in $Q(t)$. For $Q{\times}F(R)$ in response to the stimulus change (Supplementary Fig. 5f), an increase in $Q(t)$ (red to purple lines) results in a large shift in the intersection point towards the right meaning a large positive increase in $R(t)$ (orange arrow). However, for decreasing $Q(t)$ (red to green lines), the skewness of $G(R)$ brought about by small K_I results only in a minute shift of the intersect towards the left, indicating a small (almost no) negative response in $R(t)$ (cyan arrow). For high K_I ($K_\mathrm{I} = 0.1$), the behavior is intermediate between the former two cases, and the asymmetry in the output $R(t)$ becomes weak (Supplementary Fig. 5h and i). To summarize, the ‘rectified’ response is based on near zero change in R to decrease in ratio $Q = A/I$ and that is due to the ‘hemi-‘ zero-order sensitivity (i.e. only the reverse reaction of the push-pull network operates near the zero-order kinetics).

Other possible routes for rectification

The observed Ras response in cells exposed to traveling wave stimulus of cAMP predicts a rectification mechanism that filters out temporally decreasing signal stimuli. Earlier works have experimentally tested and demonstrated various aspects of the LEGI-framework such adaptive temporal and spatial sensing [4] and its response to complex stimuli[1,5], local production of the inhibitor [6] and incoherent feed-forward type network topology[7]. Our analysis suggests that rectification is separable from downstream amplification[1] and/or excitable[8] circuit thus arises at or very close to the level of LEGI -like circuitry. We introduced ultra-sensitive LEGI

to study the effect of strong nonlinearity independent of amplification. Nevertheless, chemotactic signaling pathways are highly redundant, and many details await future experimental analysis. Here, to survey other possible implementation of rectification, we analyzed earlier models to study how the required filtering characteristic as clarified by the ultra-sensitive LEGI model can be embedded.

(i) *Levchenko-Iglesias Model A* (Fig. 3A in Levchenko & Iglesias, Biophys. J. 2002 [9])

Levchenko and Iglesias (2002)[9] proposed a circuit that integrates adaptation and signal amplification in a single layer of signal transduction (Supplementary Fig. 8a). The activator '*A*' and the inhibitor '*I*' are governed by equation (1), whereas the output '*R*' (*R*) and its precursor 'R_{inactive}' (R_{in}) obey the following rate equations.

$$\frac{dR}{dt} = k_A A R_{in} - k_I I R$$
$$\frac{dR_{in}}{dt} = \sigma A - \rho I R_{in} - k_A A R_{in} + k_I I R$$

Here, the output *R* is further amplified because 'R_{inactive}' is replenished by '*A*' (the first term in the RHS of the second equation), hence the output '*R*' is no longer mass conserved. As discussed in Ref [9], for a given $Q = A/I$, stationary value of *R* follows $R = (\sigma k_A/\varrho k_I)\times Q^2$ (Supplementary Fig. 8b), meaning that amplification is in the second order thus nonlinearity is weak to support rectified response. As expected from the *R*-*Q* curve, both the positive and negative response of *R* occurs to spatially uniform change of the stimulus *S* (Supplementary Fig. 8c). In line with this, the directional response appears not only in the wavefront but in the waveback (Supplementary Fig. 8d). For comparison, the parameter values associated with *A* and *I* are the same in the basic and ultrasensitive LEGI (Supplementary Table 1). Other parameters are $k_A = 3.0$, $k_I = 1.6$, $\sigma = 3.0$, and $\varrho = 4.5$. The model has an architecture similar to the ultra-sensitive LEGI; i.e. it introduces nonlinearity to the basic LEGI scheme in the same layer of signaling cascade, however due to its weak nonlinearity it does not support rectification.

(ii) *Levchenko-Iglesias Model B* (Fig. 3B in Levchenko & Iglesias, Biophys. J. 2002 [9])

The other nonlinear scheme proposed by Levchenko and Iglesias (2002)[9] integrates adaptation and amplification by connecting in sequence the basic LEGI module and a positive feedback signal-amplification module (Supplementary Fig. 8e). The kinetics of

'A', 'I', and 'R' obey the basic LEGI model with $F(R) = k_A(R_{tot} - R)$ and $G(R) = k_I R$ (equation (1)). Downstream of the basic LEGI circuit lies another module governed by

$$\frac{dT}{dt} = k_T \frac{RT_{in}}{K_T + T_{in}} - k_\rho T$$
$$\frac{dT_{in}}{dt} = -k_T \frac{RT_{in}}{K_T + T_{in}} + k_\rho T + \sigma + k_\sigma T - \gamma_\sigma T_{in}$$

Here, the final output 'T' is activated by 'R' from the inactive state T_{in}. Furthermore, T positively regulates production of T_{in}, thereby providing a positive feedback loop that amplifies T (Supplementary Fig. 8e). The stationary response curve of T for a given $Q = A/I$ is

$$T = \frac{k_\rho k_T \gamma_\sigma + k_\rho \sigma - k_T k_\sigma R(Q) - \sqrt{\left(k_\rho k_T \gamma_\sigma + k_\rho \sigma - k_T k_\sigma R(Q)\right)^2 + 4K_T \sigma R(Q)}}{2k_\rho k_\sigma}$$

where $R(Q)$ follows $R(Q) = k_A R_{tot} Q/(\ k_A R_{tot}\ Q + k_I)$. When σ is small and k_σ is large, the relationship between Q and T realizes the characteristic rectification curve as shown in Supplementary Figure 8f ($k_T = 1.1$, $K_T = 7.5$, $k_\rho = 1.5$, $\sigma = 0.03$, $k_\sigma = 50$, $\gamma_\sigma = 9.5$) similar to that for the ultrasensitive LEGI (see Fig. 6c and Supplementary Fig. 5k for comparison). Accordingly, the model simulation shows the rectified response to spatially uniform stimulation (Supplementary Fig. 8g). To the wave stimulation, the response T was elevated at the side facing the higher concentration of S (T_+) only when S is rising in time (Supplementary Fig. 8h). Although the model can implement rectification, due to additional layers of regulation, the timescale of final output T (which should correspond to Ras activity) may deviate from the timescale of A and I (dictated by $(\gamma_i^{-1} - \gamma_a^{-1})$). As discussed in the main text, the timescale of temporal sensing estimated from directionality of cell migration suggests a close match with that of transient Ras activation.

(iii) *Amplified LEGI model* (Wang et al., Sci. Sig. 2012[1])

Based on the observations that localized PIP3 synthesis and the resulting PH-domain protein localization are strongly amplified with respect to the imposed gradient steepness, the amplified LEGI model [1] extends the basic LEGI circuit with an additional downstream amplification reaction (Supplementary Fig. 8i). The model scheme is similar to that of *Levchenko-Iglesias Model B*[9] (Supplementary Fig. 8e). The variables 'A', 'I' and 'R'

follow the basic LEGI model with $F(R) = k_A(R_{tot} - R)$ and $G(R) = k_I R$ (equation (1)). Downstream of the basic LEGI module lies a switching module that amplifies R as follows

$$\frac{\mathrm{d}Amp}{\mathrm{d}t} = k_{amp} \frac{R\left(Amp_{tot} - Amp\right)}{K_{Amp} + (Amp_{tot} - Amp)} - k_{\rho} \frac{Amp_{tot}}{K_{\rho} + Amp}.$$

For small K_{ρ}, the stationary relationship between Amp and $Q = A/I$ obeys the characteristic rectifying curve (Supplementary Fig. 8j; $R_{tot} = 2.0$, $k_A = 3.0$, $k_I = 1.6$, $Amp_{tot} = 2.0$, $k_{Amp} = 2.2$, $K_{Amp} = 1.6$, $k_{\rho} = 2.4$, and $K_{\rho} = 0.02$) similar to that shown for the ultrasensitive LEGI (see Fig. 6c and Supplementary Fig. 5k for comparison). Rectified response appeared to the uniform stimulation (Supplementary Fig. 8k). The variable Amp behaves in the rectifying manner in the wave stimulation as shown in Supplementary Figure 8l. Note that relationship between R and Amp at the stationary state is similar to that between Q and R in the ultra-sensitive LEGI model. In the amplified LEGI model, $R(Q) = k_A R_{tot} Q/(k_A R_{tot} Q + k_I)$ (Supplementary Fig. 8j, dotted line), indicating that the rectification curve at the level of the final output '*Amp*' (Supplementary Fig. 8j, solid line) is stretched out much towards higher Q. Thus, compared to the ultra-sensitive LEGI model, the response is less sensitive, and the dynamic range is more restricted. As a result, the amplified LEGI model requires careful parameter tuning to achieve rectification.

(iv) Balanced inactivation model (Levine et al., Proc. Nat. Acad. 2006 [2])

An alternative LEGI-like scheme is proposed by Levine et al. (2006)[2] which describes adaptation and amplification of chemotactic response by balanced inactivation of two signaling components. In the model, signal '*S*' activates '*A*' at the plasma membrane and '*B*' in the cytosol. '*B*' is subsequently translocated to the plasma membrane ('B_m') thereby forming a complex with '*A*' (Supplementary Fig. 8m). The governing equations are

$$\frac{dA}{dt} = k_A S - \gamma_A A - k_{AB} A B_m$$
$$\frac{dB}{dt} = k_B S - k_M B$$
$$\frac{dB_m}{dt} = k_M B - \gamma_B B_m - k_{AB} A B_m$$

'*B*' is assumed to diffuse throughout the cell, thus after its membrane translocation, 'B_m' effectively acts as the global inhibitor. The model follows the basic scheme of adaptation supported by an incoherent feedforward circuit as in LEGI; '*A*' and 'B' are both activated

by signal 'S'. A is subsequently suppressed by B with some delay incurred by its membrane translocation. The original analysis[2] studied a case $k_A = k_B$ and $k_A k_{AB} S/\gamma_A \gamma_B \gg 1$. In the following analysis, to satisfy the latter condition, we assume that k_{AB} is large without loss of generality. The stationary value of A for fixed S and B is given by

$$A = \frac{k_{AB}(k_A S - k_M B) - \gamma_A \gamma_B + \sqrt{(k_{AB}(k_A S - k_M B) - \gamma_A \gamma_B)^2 + 4k_{AB} k_A \gamma_A \gamma_B S}}{2\gamma_A k_{AB}}$$

$$\approx \frac{k_{AB}(k_A S - k_M B) + \sqrt{k_{AB}^2 (k_A S - k_M B)^2 + 4k_{AB} k_A \gamma_A \gamma_B S}}{2\gamma_A k_{AB}}.$$

At the fixed point, $k_B S = k_M B$ holds, thus $k_A S - k_M B$ vanishes when $k_A = k_B$, hence $A \approx \sqrt{k_A \gamma_B / k_{AB} \gamma_A} \sqrt{S}$. The change in the basal level of A is negligibly small compared to the signal response, because the value of A at the stationary state is in the order of $k_{AB}^{-1/2}$ [2]. During the transient response, $k_A S - k_M B$ is non-zero, and its contribution to A appears at zero-th order of k_{AB}.

$$A \approx \frac{k_A S - k_M B + \sqrt{(k_A S - k_M B)^2}}{2\gamma_A} = \begin{cases} (k_A S - k_M B)/\gamma_A & k_A S - k_M B \geq 0 \\ 0 & otherwise \end{cases}$$

In the balanced inactivation model, plotting A as a function of $q \equiv k_A S - k_M B$, serves the equivalent of the R-Q curve in the LEGI models (Supplementary Fig. 8n; $k_A = k_B = 1.5$, $\gamma_A = 0.1$, $\gamma_B = 0.02$, $k_{AB} = 1000$, and $k_M = 0.75$). Because $q = k_A S - k_M B$ is positive for increasing stimuli and negative for decreasing stimuli, response in A appears only for temporally increasing signal input and not for temporally decreasing input (Supplementary Fig. 8o). Supplementary Figure 8p shows results of the numerical simulations of the model in the wave stimulation. In the simulation, B is assumed to diffuse at D = 30 μm^2 sec^{-1}. The response A shows strong directional response only when S is increasing in time.

While these results indicate that the balanced-inactivation model [2] is capable of rectification (Supplementary Fig. 8m-p), there were some disagreements with the experimental observations. For the spatially uniform concentrations of S that is decreasing in time, A always decline monotonically regardless of parameter values. Therefore, the model was not able to reproduce the pulsatile negative response to decreasing stimuli observed in weakly starved cells (Fig. 7b). Also, the response amplitude in the balanced-inactivation model shows strong dependence to the input level which is unlike the response observed for RBD

localization.

Cell migration

By employing a phenomenological description of cell movement coupled to directional sensing, let us confirm how experimentally observed directionality of cell displacement could be brought about. Although vastly oversimplified, we shall assume the velocity of a cell, $\mathrm{d}x(t)/\mathrm{d}t = v(t)$ correlates with the difference of the response R between both sides of the cell; namely,

$$\mu \frac{\mathrm{d}v(t)}{\mathrm{d}t} = -v(t) + h(R_+) - h(R_-), \quad (1)$$

$$h(R) = v_0 R^2 / \left(R^2 + \Theta^2\right). \qquad (2)$$

Here, μ defines the relaxation time of the cell motion and $h(R)$ governs dependency of cell motion on R, for which we adopt a simple saturating function. For numerical simulations of equation (2) and (3) and Supplementary equations (1) and (2) for the wave stimulus, parameters are set to μ = 25 sec, v_0 = 12 μm min^{-1}, and Θ = 0.2.

For sufficiently small wave transit time (Supplementary Fig. 6e, 1 min/L_P), cell motion is almost undetectable. As the transit time is increased, directional migration in the positive direction is observed (Supplementary Fig. 6f, 7 min/L_P). A further increase in the transit time results in reduction of the mean velocity of cell migration due to cancelation of forward movement in the wavefront by the reverse movement in the waveback (Supplementary Fig. 6g, 26 min/L_P; Supplementary Fig. 6h, 35 min/L_P). These results provide us with an intuitive description of the limited range of transit time where rectified directional migration can occur (Fig. 1g). Moreover, as shown in Fig. 1g and Supplementary Fig. 6h, the total displacement at large transit time becomes negative, because the cells migrate in the waveback by following the trailing end. To be exact, relative speed between the cells and the signal propagating at the velocity V_S is given by $V_S + v$ in the wavefront and $V_S - v$ in the waveback (Doppler shift), thus cells moving in the same direction as the signal wave spend longer time sensing the gradient than those moving against the wave (Supplementary Fig. 6h, green). When the wave is slow enough, i.e. in the stationary directional sensing scheme, this difference results in the negative net displacement (Supplementary Fig. 6h).

Supplementary References

1. Wang, C. J., Bergmann, A., Lin, B., Kim, K. & Levchenko, A. Diverse sensitivity thresholds in dynamic signaling responses by social amoebae. *Sci Signal* **5,** ra17–ra17 (2012).
2. Levine, H., Kessler, D. A. & Rappel, W.-J. Directional sensing in eukaryotic chemotaxis: a balanced inactivation model. *Proc Natl Acad Sci USA* **103,** 9761–9766 (2006).
3. Goldbeter, A. & Koshland, D. E. An amplified sensitivity arising from covalent modification in biological systems. *Proc Natl Acad Sci USA* **78,** 6840–6844 (1981).
4. Janetopoulos, C., Ma, L., Devreotes, P. N. & Iglesias, P. A. Chemoattractant-induced phosphatidylinositol 3,4,5-trisphosphate accumulation is spatially amplified and adapts, independent of the actin cytoskeleton. *Proc Natl Acad Sci USA* **101,** 8951–8956 (2004).
5. Ma, L., Janetopoulos, C., Yang, L., Devreotes, P. N. & Iglesias, P. A. Two complementary, local excitation, global inhibition mechanisms acting in parallel can explain the chemoattractant-induced regulation of PI(3,4,5)P3 response in dictyostelium cells. *Biophys J* **87,** 3764–3774 (2004).
6. Xu, X., Meier-Schellersheim, M., Yan, J. & Jin, T. Locally controlled inhibitory mechanisms are involved in eukaryotic GPCR-mediated chemosensing. *J Cell Biol* **178,** 141–153 (2007).
7. Takeda, K. *et al*. Incoherent feedforward control governs adaptation of activated ras in a eukaryotic chemotaxis pathway. *Sci Signal* **5,** ra2 (2012).
8. Huang, C.-H., Tang, M., Shi, C., Iglesias, P. A. & Devreotes, P. N. An excitable signal integrator couples to an idling cytoskeletal oscillator to drive cell migration. *Nat Cell Biol* **15,** 1307–1316 (2013).
9. Levchenko, A. & Iglesias, P. A. Models of eukaryotic gradient sensing: application to chemotaxis of amoebae and neutrophils. *Biophys J* **82,** 50–63 (2002).
